# Costs and Benefits of Transgenerational Induced Resistance in Arabidopsis

**DOI:** 10.3389/fpls.2021.644999

**Published:** 2021-02-26

**Authors:** Ana López Sánchez, David Pascual-Pardo, Leonardo Furci, Michael R. Roberts, Jurriaan Ton

**Affiliations:** ^1^Plant Production and Protection (P3) Centre, Institute for Sustainable Food, Department of Animal and Plant Sciences, The University of Sheffield, Sheffield, United Kingdom; ^2^Lancaster Environment Centre, Lancaster University, Lancaster, United Kingdom

**Keywords:** Arabidopsis, transgenerational effects, induced resistance, costs and benefits, transgenerational phenotypic plasticity

## Abstract

Recent evidence suggests that stressed plants employ epigenetic mechanisms to transmit acquired resistance traits to their progeny. However, the evolutionary and ecological significance of transgenerational induced resistance (t-IR) is poorly understood because a clear understanding of how parents interpret environmental cues in relation to the effectiveness, stability, and anticipated ecological costs of t-IR is lacking. Here, we have used a full factorial design to study the specificity, costs, and transgenerational stability of t-IR following exposure of *Arabidopsis thaliana* to increasing stress intensities by a biotrophic pathogen, a necrotrophic pathogen, and salinity. We show that t-IR in response to infection by biotrophic or necrotrophic pathogens is effective against pathogens of the same lifestyle. This pathogen-mediated t-IR is associated with ecological costs, since progeny from biotroph-infected parents were more susceptible to both necrotrophic pathogens and salt stress, whereas progeny from necrotroph-infected parents were more susceptible to biotrophic pathogens. Hence, pathogen-mediated t-IR provides benefits when parents and progeny are in matched environments but is associated with costs that become apparent in mismatched environments. By contrast, soil salinity failed to mediate t-IR against salt stress in matched environments but caused non-specific t-IR against both biotrophic and necrotrophic pathogens in mismatched environments. However, the ecological relevance of this non-specific t-IR response remains questionable as its induction was offset by major reproductive costs arising from dramatically reduced seed production and viability. Finally, we show that the costs and transgenerational stability of pathogen-mediated t-IR are proportional to disease pressure experienced by the parents, suggesting that plants use disease severity as an environmental proxy to adjust investment in t-IR.

## Introduction

Phenotypic plasticity allows organisms to modify their biochemical, physiological, or morphological traits to survive in changing environments (Schlichting, 1986). While phenotypic plasticity has mostly been studied within the lifespan of organisms, there is increasing evidence that life history experiences of individuals can influence traits in their progeny. These include simple direct maternal effects such as nutrient provisioning to progeny. However, some transgenerational effects can persist over multiple generations and involve heritable epigenetic changes (Lämke and Bäurle, 2017; Bošković and Rando, 2018). These epigenetic responses have the potential to provide adaptive benefits to progeny, thereby enhancing evolutionary fitness of the parents. When facing environmental changes, organisms can adopt various transgenerational strategies to optimize their fitness. When environments change frequently and are unpredictable, parents may adopt a bet-hedging strategy to increase the variability within their progeny (Crean and Marshall, 2009). By contrast, when environments undergo directional and stable changes, which present a more predictable cue about future environmental conditions, parents could enhance reproductive fitness by transmitting specific adaptive traits to their progeny (Marshall and Uller, 2007; Proulx and Teotonio, 2017).

Transgenerational responses to stress have been reported in both plants and animals, ranging from maladaptive pathological effects of environmental pollutants to adaptive immunological traits that increase disease resistance (Holeski et al., 2012; Rasmann et al., 2012; Perez and Lehner, 2019; Tetreau et al., 2019). In plants, the latter response has been referred to as “transgenerational acquired resistance” or “transgenerational induced resistance (t-IR),” which is typically based on a sensitization, or “priming,” of the immune system, mediating a faster and/or stronger immune response (Wilkinson et al., 2019). We have previously demonstrated that bacterial speck disease, caused by the hemi-biotrophic pathogen *Pseudomonas syringae* pv. *tomato* (*Pst*), results in t-IR that can be maintained over two stress-free generations in the self-fertilizing annual plant Arabidopsis (*Arabidopsis thaliana*) (Luna et al., 2012; Stassen et al., 2018). Although the exact epigenetic mechanisms underpinning t-IR are still under investigation, the induction and/or transmission of the response requires DNA demethylation at transposable elements and is associated with genome-wide changes in DNA methylation (Luna and Ton, 2012; Lopez Sanchez et al., 2016; Stassen et al., 2018; Furci et al., 2019). These results are supported by numerous other studies that have reported transgenerational changes in DNA methylation in response to environmental stress (Lämke and Bäurle, 2017; Wilkinson et al., 2019).

Evolutionary models predict that parental effects on specific traits can act as an adaptive mechanism to increase fitness in changeable environments (Leimar and McNamara, 2015; Pigeault et al., 2016; Proulx and Teotonio, 2017). However, despite numerous reports of transgenerational effects of stress in plants, there is still controversy over whether these responses are adaptive (Uller et al., 2013; Burggren, 2015; Crisp et al., 2016). Transgenerational phenotypic responses to light and water availability have been shown to provide improved fitness when the environments of parents and progeny are matched (Galloway and Etterson, 2007; Herman et al., 2012). However, when parent and progeny environments are mismatched, transgenerational effects can be unfavorable, which may explain why many epigenetic modifications are erased during sexual reproduction (Iwasaki and Paszkowski, 2014; Crisp et al., 2016; Gehring, 2019).

In the case of plant defense against herbivores and disease, the associated costs on host plants are often assumed to have driven the evolution of inducible defenses (Zangerl, 2003; Karasov et al., 2017). Costs associated with induced defense include direct allocation costs arising from the biosynthesis of defensive proteins and secondary metabolites (Züst et al., 2011). Priming of inducible defense provides a mechanism to optimize the cost–benefit trade-offs of defense, by preserving inducible defenses in a state of readiness for augmented responses to future infection without incurring direct costs of maintaining active defense (Karasov et al., 2017; Wilkinson et al., 2019). As well as direct allocation costs, ecological costs are evident when defenses targeted against pathogens or herbivores have negative consequences for interactions with beneficial microbes (Ruiz-Lozano et al., 1999) or pollinators (Strauss et al., 1999). Ecological costs also arise when increased resistance against one form of biotic attack is associated with increased susceptibility against another (Vos et al., 2013). In the case of pathogen-mediated t-IR, progeny from *P. syringae*-infected Arabidopsis expressed t-IR against another biotrophic pathogen, *Hyaloperonospora arabidopsidis* (*Hpa*), but the same progeny showed enhanced susceptibility to the necrotrophic fungus *Alternaria brassicicola* (Luna et al., 2012). Similarly, progeny from spider mite-infested Arabidopsis were primed to resist spider mites and aphids but suffered increased susceptibility to *P. syringae* (Singh et al., 2017). These results suggest that t-IR carries both benefits and costs in terms of resistance in matched and mismatched environments, respectively. However, a comprehensive understanding about the reciprocal costs and benefits of t-IR in terms of changes in (a)biotic stress resistance is lacking. Similarly, the relationship between stress severity experienced by the parents, on the one hand, and intensity and transgenerational stability of t-IR, on the other hand, remains unknown. Hence, a better understanding of the dose-dependent effects of (a)biotic stress on the costs and benefits of t-IR is required to understand the ecological significance of this epigenetic plant response.

In considering the evolution of adaptive parental effects, various studies have highlighted the importance of full factorial experimental designs to simultaneously assess the associated costs and benefits (Marshall and Uller, 2007; Bonduriansky et al., 2012; Uller et al., 2013; Burgess and Marshall, 2014; Tetreau et al., 2019). However, to date, most studies in plants have focused on the underpinning mechanisms and have overlooked the evolutionary and ecological significance of t-IR. Here, we employ a full factorial reciprocal experimental design to systematically test the costs and benefits of t-IR within a single experimental framework. We have examined the specificity of t-IR by quantifying impacts of three parental stresses at different levels of intensity in both matched and mismatched progeny environments. The results provide evidence that plants can adjust the strength and/or durability of t-IR based on their ability to interpret the reliability of predictive cues from their environment.

## Materials and Methods

### Plant Material and Growth Conditions

**FIGURE 1:**
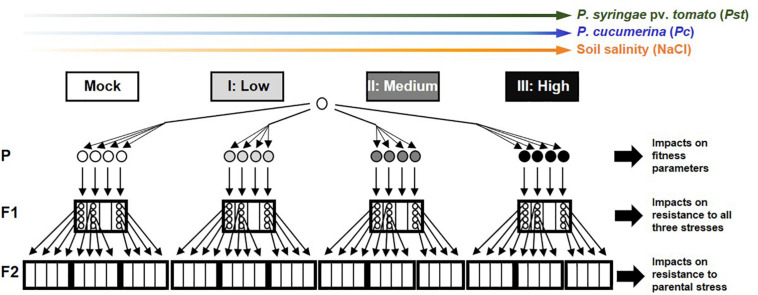
Full factorial experimental design of the study. *Arabidopsis thaliana* plants (accession Col-0) from a common ancestor were exposed to increasing stress intensities (Mock, Low, Medium, and High) by the (hemi)biotrophic bacterial pathogen *Pseudomonas syringae* pv. *tomato* (green), the necrotrophic fungal pathogen *Plectosphaerella cucumerina* (blue), or soil salinity (NaCl; orange). Plants in this parental generation (P) were evaluated for impacts on fitness parameters. Four plants per stress level were selected to generate F1 populations, which were analyzed for transgenerational changes in resistance against all three stresses, in order to determine the specificity of transgenerational induced resistance (t-IR), potential costs arising from increased susceptibility, and dose-dependency of t-IR intensity on parental stress. For each parental treatment, four individual plants from three independent F1 populations were randomly selected to set seed in the absence of stress. The resulting F2 populations were analyzed for resistance against the parental stress to examine dose-dependent effects on t-IR stability. Circles indicate individual plants; small (thin-lined) boxes indicate F1/F2 populations derived from a common ancestor in the previous generation; big (bold-lined) boxes indicate pooled F1/F2 populations from a common ancestor two generations earlier.

All *A. thaliana* lines described in this study are in the genetic background of accession Col-0 (NCBI, Tax ID 3702). To exclude confounding effects of t-IR from stress in previous generations, all lines originated from a common ancestor of a population that had maintained under stress-free conditions (mock-inoculated) in two previous generations (Luna et al., 2012). Except for the stress treatments, all plants were grown under similar conditions (see Supplementary Methods for details). To generate F1 populations, six to eight parental plants of 4.5 weeks old were subjected to mock/stress treatments over a duration of 3 weeks, after which four parental plants with representative symptoms were moved to long-day conditions (16 h light/8 h darkness) to set seed and generate F1 populations (Figure 1). Within three F1 populations from each parental treatment, four plants were kept apart to set seed under stress-free conditions. This resulted in four F2 populations that were derived from one parent plant and a total of 12 F2 populations per parental treatment (Figure 1). Details of all F1 and F2 populations are presented in Supplementary Table 1.

### Stress Treatments of Parental Plants

Inoculation with biotrophic *P. syringae* pv. *tomato* (*Pst*) was performed at 3–4-day intervals over a total period of 3 weeks, as detailed in the Supplementary Methods. Plants were subjected to different *Pst* disease pressures: no disease (Mock; six subsequent inoculations with the mock suspension), low disease (*Pst*-I; two inoculations with *Pst* followed by four mock inoculations), medium disease (*Pst*-II; four inoculations with *Pst* followed by two mock inoculations solution), and high disease (*Pst*-III; six subsequent inoculations with *Pst*). To ensure necrotrophic infection by *Plectosphaerella cucumerina* (*Pc*), inoculation was performed by placing 6 μl-droplets (10^6^ spores/ml) onto fully expanded leaves of approximate similar age (Petriacq et al., 2016), as detailed in the Supplementary Methods. Plants were subjected to different *Pc* disease pressures: no disease (Mock; six leaves were mock-inoculated), low disease (*Pc*-I; two leaves *Pc*-inoculated and four leaves mock-inoculated), medium disease (*Pc*-II; four leaves *Pc*-inoculated and two leaves mock-inoculated), and high disease (*Pc*-III; six leaves *Pc*-inoculated). After inoculation, plants were kept at 100% RH for 2 weeks until visible disease symptoms appeared in >80% of the leaf surface (necrosis and chlorosis). To prevent sporulation and ongoing disease progression, plants were returned to 60% RH before moving to long-day conditions 1 week later. Salt stress was applied by soil-drenching with 100 mM NaCl solution. Plants were subjected to different stress levels over the 3-week period: mock treatment (S-I; drenched 6× with water), low stress (S-II; drenched 2× with NaCl and 4× with water), medium stress (S-III; drenched 4× with NaCl and 2× with water), and high stress (S-III; plants drenched 6× with NaCl). Plants returned to a normal watering regime when moved to long-day conditions. Relative growth rate (RGR) was determined non-destructively by quantification of green leaf area (GLA) before and after stress treatments, as detailed in the Supplementary Methods. Reproductive fitness was estimated by seed production and seed viability, as described in the Supplementary Methods.

### Quantification of Transgenerational Resistance Phenotypes

To quantify resistance against biotrophic *Pst*, leaves of 4.5-week-old plants were spray-inoculated with a bacterial suspension (see Supplementary Methods). Bacterial growth was quantified at 3 dpi by dilution plating on selective agar plates (see Supplementary Methods). Inoculation with biotrophic *Hpa* and quantification of *Hpa* resistance was performed as described previously (Lopez Sanchez et al., 2016; see also Supplementary Methods). To quantify resistance against *Pc*, 4 leaves/plant were droplet-inoculated when plants were 4.5 weeks old and maintained at 100% RH. Resistance was quantified by average lesion diameters (see Supplementary Methods). Quantification of salt tolerance was based on root growth analysis on agar plates containing 0, 50, and 100 mM NaCl. Assays were conducted as described previously (Verslues et al., 2006; Claeys et al., 2014) with minor modifications (see Supplementary Methods).

### Statistical Analysis

Analytical statistics was performed using R studio (v 1.1.456)^1^, supporting R software (v 3.5.1)^2^. Statistical significance of treatment effects on continuous variables was analyzed by linear models; statistical significance of treatment effects on categorical variables (class frequencies) was analyzed by Fisher’s exact tests. Details about data transformations, statistical models, and R software packages are described in the Supplementary Methods.

## Results

### Dose-Dependent Effects of Three Environmental Stresses on Growth, Seed Production, and Seed Viability

**FIGURE 2:**
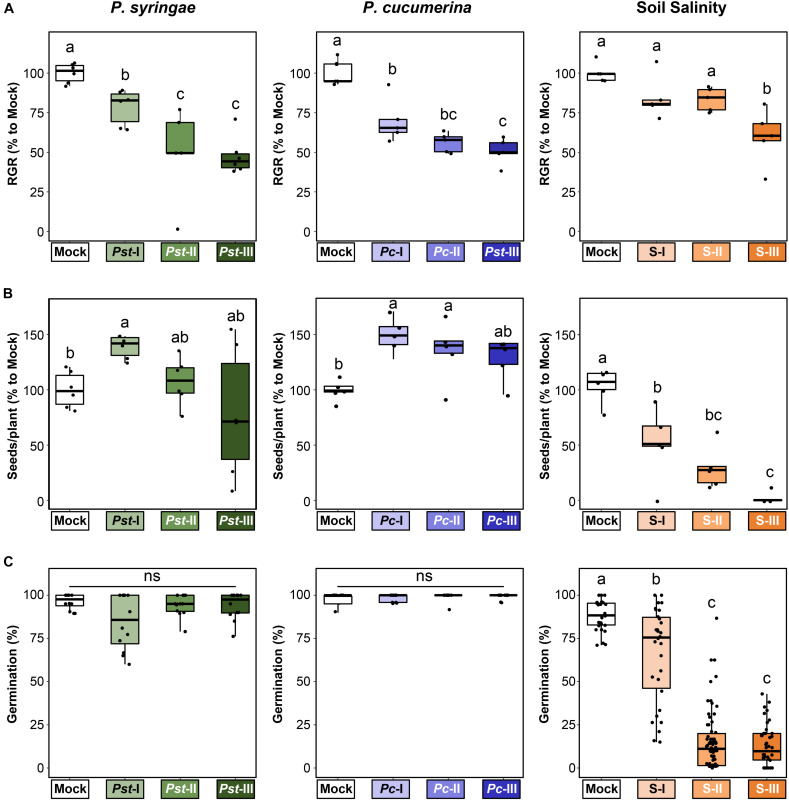
Differential impacts of three (a)biotic stresses on parental fitness parameters. Plants in the parental generation (4.5 weeks old) were exposed to varying stress intensities by *P. syringae* pv *tomato* (Mock, *Pst*-I, *Pst*-II, and *Pst*-III), *P. cucumerina* (Mock, *Pc*-I, *Pc*-II, and *Pc*-III), or soil salinity (Mock, S-I, S-II, and S-III) over a 3-week period before transferring to long-day conditions to trigger flowering and set seed. Boxplots show the interquartile range (IQR; box) ± 1.5xIQR (whiskers), including median (horizontal line) and replication units (single dots). **(A)** Impacts on relative growth rate (RGR) during the period of stress exposure. Data represent RGR values of single plants (*n* = 5–6) normalized to the average RGR of Mock-treated plants (100%). Different letters indicate statistically significant differences (ANOVA + Tukey’s *post hoc* test, α = 0.05). **(B)** Impacts on seed production. Data represent seed numbers per plant (*n* = 5–6) normalized to average value of Mock-treated plants (100%). Different letters indicate statistically significant differences (*Pst*: Welch ANOVA + Games-Howell *post hoc* test, α = 0.05; *Pc* and salt: ANOVA + Tukey’s *post hoc* test, α = 0.05). **(C)** Impacts on seed viability. Seed viability was determined 5 days after planting of surface-sterilized and stratified seeds onto 0.2× Murashige and Skoog (MS) agar plates. Data represent mean germination percentages per plate (25 seeds/plate) of seed batches from four similarly treated parents (*n* = 15–60). Different letters indicate statistically significant differences (Welch ANOVA + Games-Howell *post hoc* test; α = 0.05). Viability data for seed batches from individual plants are presented in Supplementary Figures 1A–C.

To obtain a comprehensive assessment of the costs and benefits of t-IR, we generated populations of plants descended from parents exposed to three different types of (a)biotic stress, and tested each population for resistance against those same three stresses. Starting from a single common ancestor to minimize (epi)genetic variation, we produced populations of Arabidopsis progeny that in the parental generation had been exposed to each of the three stresses: the (hemi)biotrophic pathogen *P. syringae* pv. *tomato* DC3000 (*Pst*), the necrotrophic pathogen *Pc*, and salt stress (Figure 1). For each stress type, we applied four severity levels (mock plus three increasing levels of the stress). To verify that these treatments differentially impacted the parental lines, we assessed their growth and development (Figure 2). All stresses induced a dose-dependent decline in relative growth rate (RGR), confirming that the plants perceived and responded to the stresses in a dose-dependent manner (Figure 2A). By contrast, seed production responded differently to the three stresses. The lowest levels of disease by *Pst* and *Pc* stimulated seed production, whereas the highest stress levels by these diseases had no statistically significant effect on seed production (Figure 2B). This suggests that Arabidopsis can adapt to these diseases by compensating the reduced growth during pathogen exposure with increased seed production at the end of its life cycle. Conversely, increasing levels of soil salinity caused a dose-dependent reduction in seed production (Figure 2B), indicating that Arabidopsis does not recover as efficiently from this stress as it does from disease by *Pst* or *Pc*. Similar patterns were observed for seed viability, where *Pst* and *Pc* had no significant effects (Figure 2C and Supplementary Figures 1A,B), whereas soil salinity caused a dramatic dose-dependent decline in seed viability (Figure 2C and Supplementary Figure 1C), which was absent in F2 seeds after one stress-free F1 generation (Supplementary Figure 1D).

### Parental Stress Leads to Beneficial or Neutral Impacts on Resistance of Progeny in Matched Environments

**FIGURE 3:**
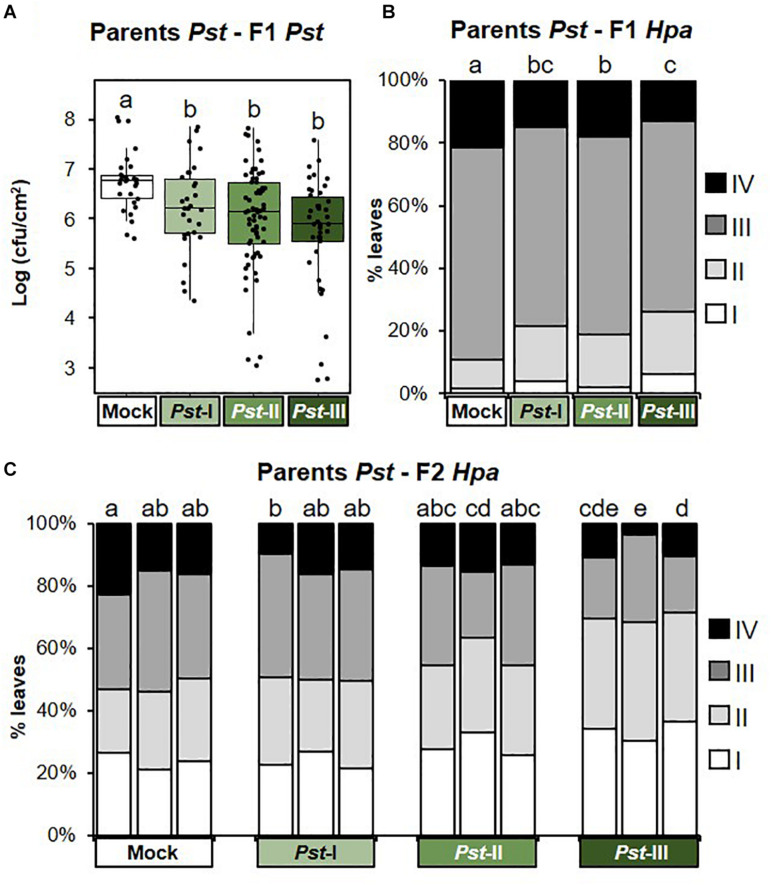
Intensity and transgenerational stability of *Pseudomonas syringae* pv. *tomato*-mediated t-IR in matched environments. Parental plants had been exposed to different disease severities by the biotrophic bacterium *Pseudomonas syringae* pv. *tomato* (*Pst*; Mock, *Pst*-I, *Pst*-II, and *Pst*-III). F1 and F2 plants were analyzed for resistance against the same pathogen (*Pst*) and/or the biotrophic Oomycete *Hyaloperonospora arabidopsidis* (*Hpa*). **(A)** t-IR against *Pst* in F1 progeny at 3 dpi. Boxplots show the interquartile range (IQR; box) ± 1.5xIQR (whiskers), including median (horizontal line) and replication units (dots). Data represent ^10^log-transformed bacterial titers (log cfu/cm^2^) in leaves of single plants within F1 populations from similarly treated parents (*n* = 42). Different letters indicate statistically significant differences (Welch ANOVA + Games-Howell test, α = 0.05). Data for individual F1 populations are shown in Supplementary Figure 2A. **(B)** t-IR against *Hpa* in F1 progeny. *Hpa* colonization was quantified at 6 dpi by assigning trypan-blue stained leaves to four *Hpa* resistance classes (I, healthy; II, hyphal colonization only; III, hyphal colonization with conidiospores; and IV, hyphal colonization with conidiospores and oospores). Stacked bars show leaf frequency distributions within F1 populations from similarly treated parental plants (*n* = 600–1,000). Different letters indicate statistically significant differences (pairwise Fisher’s exact tests + Bonferroni FDR, α = 0.05). Data for individual F1 populations are shown in Supplementary Figure 2B. **(C)** t-IR against *Hpa* in F2 progeny at 6 dpi after one stress-free F1 generation. Stacked bars show leaf frequency distributions across *Hpa* resistance classes within F2 populations that share a common parental ancestor (*n* = 300–350). Different letters indicate statistically significant differences (pairwise Fisher’s exact tests + Bonferroni FDR; α = 0.05). Data for individual F2 populations are shown in Supplementary Figure 2C.

**FIGURE 4:**
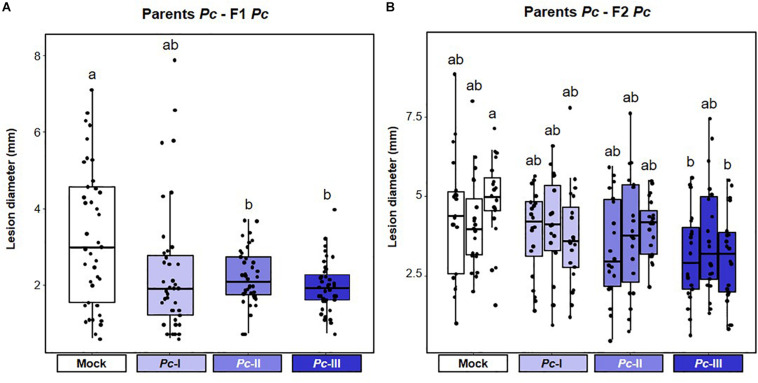
Intensity and transgenerational stability of *Plectosphaerella cucumerina*-mediated t-IR in matched environments. Parental plants had been exposed to different disease severities by necrotrophic *Plectosphaerella cucumerina* (*Pc*; Mock, *Pc*-I, *Pc*-II, and *Pc*-III). F1 and F2 plants were analyzed for resistance against the same pathogen. Lesion diameters were determined in four leaves/plant at 15 dpi and the average lesion diameter per plant was used as statistical unit of replication. Boxplots show the interquartile range (IQR; box) ± 1.5xIQR (whiskers), including median (horizontal line) and replication units (dots). **(A)** t-IR against *Pc* in F1 progeny. Data represent lesion diameters (mm) of plants within F1 populations from similarly treated parents (*n* = 40). Different letters indicate statistically significant differences (Welch ANOVA + Games-Howell test, α = 0.05). Data for individual F1 populations are shown in Supplementary Figure 3A. **(B)** t-IR against *Pc* in F2 progeny after a stress-free F1 generation. Data represent lesion diameters of plants within F2 populations that share a common parental ancestor (*n* = 20). Different letters indicate statistically significant differences (ANOVA + Tukey’s *post hoc* test; α = 0.05). Data for individual F2 populations are shown in Supplementary Figure 3B.

**FIGURE 5:**
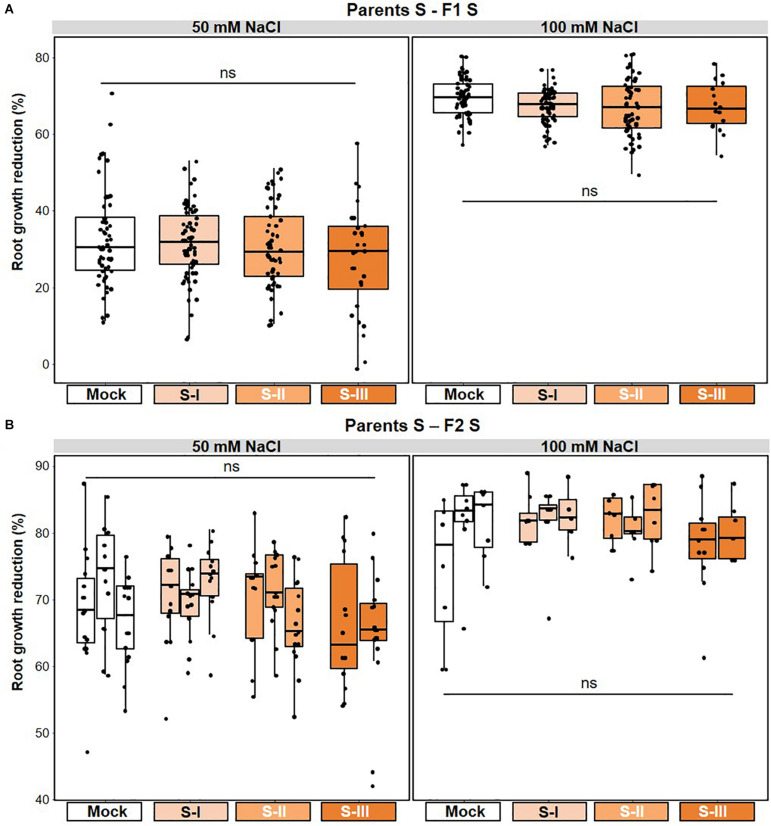
Lack of salt-mediated t-IR in matched environments. Parental plants had been exposed to different stress intensities by soil salinity (NaCl; Mock, S-I, S-II, and S-III). Salt tolerance of F1 and F2 plants was quantified by root growth reduction (%) over a 5-day period on NaCl-containing agar medium relative to the average root growth on agar medium without NaCl. Boxplots show the interquartile range (IQR; box) ± 1.5xIQR (whiskers), including median (horizontal line) and replication units (dots). **(A)** Unaltered tolerance of F1 plants to 50 and 100 mM NaCl. Data represent growth reduction percentages of single plants within F1 populations from similarly treated parents (*n* = 60). ns, no statistically significant differences (ANOVA; α = 0.05). Root growth data for individual F1 populations are shown in Supplementary Figure 4A; root tolerance data for individual F1 populations are shown in Supplementary Figure 4C. **(B)** Unaltered tolerance of F2 plants to 50 and 100 mM NaCl after one stress-free F1 generation. Data represent growth reduction percentages of single plants within F2 populations that share a common parental ancestor (*n* = 18–20). ns, no statistically significant differences (ANOVA; α = 0.05). Root growth data for individual F2 populations are shown in Supplementary Figure 4B; root tolerance data for individual F2 populations are shown in Supplementary Figure 4D.

Next, we investigated t-IR in F1 progeny against the same stress to which the parents had been exposed (matched environments). Parents exposed to disease by biotrophic *Pst* produced F1 progeny that were more resistant to both *Pst* (Figure 3A and Supplementary Figure 2A), and the biotrophic Oomycete *Hpa* (Figure 3B and Supplementary Figure 2B). These findings support our previous results (Luna et al., 2012) and demonstrate that t-IR by *Pst* is not specific at the level of pathogen species, but that it protects against taxonomically unrelated pathogens with similar biotrophic lifestyles. To determine whether Arabidopsis can also develop t-IR after infection by necrotrophic pathogens, we tested F1 progeny from parents exposed to increasing disease by the necrotrophic fungus *Pc* for resistance against the same pathogen. Compared to progeny from mock-inoculated parents, all but the lowest severity of parental disease resulted in a statistically significant suppression of *Pc* lesion development in F1 progeny (Figure 4A and Supplementary Figure 3A). Hence, necrotrophic *Pc* elicits t-IR that is effective in matched environments. Finally, we investigated the transgenerational effects of soil salinity in matched environments. To this end, F1 progeny from parents exposed to increasing NaCl concentrations in the soil were analyzed for root growth inhibition on agar medium supplemented with 50 and 100 mM NaCl, which is a common method to quantify salt tolerance in Arabidopsis (Verslues et al., 2006; Claeys et al., 2014). F1 populations from differently treated parents showed small but statistically significant differences in root growth on agar medium containing 0 and 50 mM NaCl (Supplementary Figure 4A), which were absent in the F2 generation (Supplementary Figure 4B). However, relative to plates with no addition of salt, the degree of inhibition of root growth caused by inclusion of 50 or 100 mM NaCl was similar between populations from all parental treatments (Figures 5A,B and Supplementary Figures 4C,D). Thus, under our conditions, progeny from salt-stressed plants fail to develop t-IR in matched environments.

### The Costs of t-IR Become Apparent in Mismatched Environments

**FIGURE 6:**
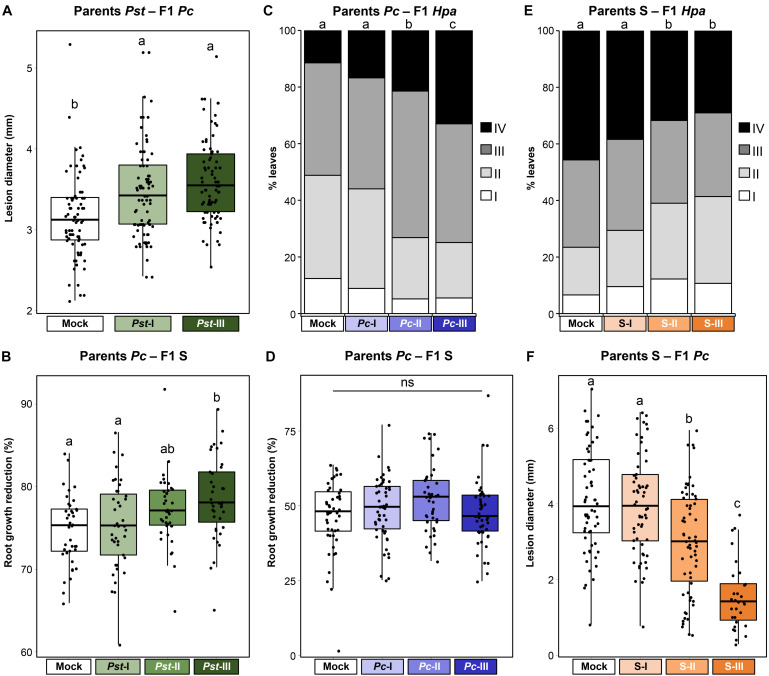
Costs and benefits of t-IR in mismatched environments. Parental plants had been exposed to different stress severities by *Pst* (green), *Pc* (blue), or soil salinity (orange). F1 plants were tested for resistance against different stresses than the parental stress. **(A)** Increased *Pc* susceptibility in F1 progeny from *Pst*-exposed parents. Box plots show lesion diameters (mm) of plants within F1 populations from similarly treated parental plants (*n* = 76–80). See the legend of Figure 4 for details. Different letters indicate statistically significant differences between parental treatments (ANOVA + Tukey’s *post hoc* test; α = 0.05). Data for individual F1 populations are shown in Supplementary Figure 5A. **(B)** Reduced salt tolerance in F1 progeny from *Pst*-exposed parents. Box plots show root growth reduction percentages by 50 mM NaCl of plants within F1 populations from similarly treated parental plants (*n* = 40). See the legend of Figure 5 for details. Different letters indicate statistically significant differences (ANOVA + Tukey’s *post hoc* test; α = 0.05). Root growth data for individual F1 populations at 0, 50, and 100 mM NaCl are shown in Supplementary Figure 5B; tolerance data for individual F1 populations to 50 and 100 mM NaCl are shown in Supplementary Figure 5C. **(C)** Increased *Hpa* susceptibility in F1 progeny from *Pc*-exposed parents. Stacked bars show leaf frequency distributions across *Hpa* resistance classes within F1 populations from similarly treated parents (*n* = 400–500). See the legend of Figure 3B for details. Different letters indicate statistically significant differences (pairwise Fisher’s exact tests + Bonferroni FDR, α = 0.05). Data for individual F1 populations are shown in Supplementary Figure 6A. **(D)** Unaltered salt tolerance in F1 progeny from *Pc*-exposed parents. Box plots show root growth reduction percentages by 50 mM NaCl of plants within F1 populations from similarly treated parental plants (*n* = 47). See the legend of Figure 5 for details. ns, no statistically significant differences (ANOVA; α = 0.05). Root growth data for individual F1 populations at 0, 50, and 100 mM NaCl are shown in Supplementary Figure 6B; tolerance data for individual F1 populations to 50 and 100 mM NaCl are shown in Supplementary Figure 6C. **(E)** Non-specific t-IR against *Hpa* in F1 progeny from NaCl-exposed parents. Stacked bars show leaf frequency distributions across *Hpa* resistance classes within F1 populations from similarly treated parents (*n* = 350–800). See the legend of Figure 3B for details. Different letters indicate statistically significant differences (pairwise Fisher’s exact tests + Bonferroni FDR, α = 0.05). Data for individual F1 populations are shown in Supplementary Figure 7A. **(F)** Non-specific t-IR against *Pc* in F1 progeny from NaCl-treated parents. Box plots show lesion diameters (mm) of plants within F1 populations from similarly treated parental plants (*n* = 30–60). See the legend of Figure 4 for details. Different letters indicate statistically significant differences between parental treatments (ANOVA + Tukey’s *post hoc* test; α = 0.05). Data for individual F1 populations are shown in Supplementary Figure 7B.

To investigate the resistance phenotypes of our F1 populations against stresses other than the parental stress (mismatched environments), we used a reciprocal experimental design based on the three parental stress treatments (Figure 1). We have previously reported that *Pst*-mediated t-IR is associated with increased susceptibility to the necrotrophic fungus *A. brassicicola* (Luna et al., 2012). In agreement with this finding, F1 progeny from *Pst*-infected plants developed larger lesions after inoculation with necrotrophic *Pc* (Figure 6A and Supplementary Figure 5A). Furthermore, F1 progeny from *Pst*-exposed parents showed a statistically significant increase in root growth inhibition by 50 mM NaCl (Figure 6B and Supplementary Figures 5B,C), indicating increased sensitivity to salt stress. Next, we investigated F1 progeny from *Pc*-infected parents for resistance against biotrophic *Hpa* and salt stress. F1 populations from parents exposed to the two highest severities of *Pc* disease showed increased susceptibility to *Hpa* (Figure 6C and Supplementary Figure 6A) but were unaffected in salt tolerance (Figure 6D and Supplementary Figures 6B,C). Together, our results indicate that the potential benefits of t-IR by pathogens are traded off against costs of increased susceptibility to other stresses that become apparent in mismatched environments. In that regard, it was surprising that F1 progeny from parents exposed to the highest degrees of soil salinity showed increased resistance to both biotrophic *Hpa* and necrotrophic *Pc* (Figures 6E,F and Supplementary Figures 7A,B). However, the ecological significance of this non-specific t-IR by parental salt stress must be considered against the lack of beneficial effects in the matched environment (Figure 5), as well as the severe fitness costs arising from reduced plant growth, seed production, and seed viability (Figure 2).

### Stress Intensity Acts as a Weighted Indicator for t-IR Investment

Since t-IR is associated with costs in mismatched environments, we considered the possibility that plants can adjust t-IR investment in accordance with the reliability of the environmental stress signal. We hypothesized that severe stress is perceived as a more reliable predictor of the progeny environment, resulting in stronger t-IR investment. In matched environments, t-IR by *Pst* and *Pc* was strongest in F1 progeny from parents exposed to the highest stress levels, although the difference in intensity between the lowest and highest parental stress intensities was not statistically significant (Figures 3A,B,4A and Supplementary Figures 2A,B, 3A). Similarly, in mismatched environments, F1 progeny from parents exposed to the highest level of *Pst* disease showed a greater degree of susceptibility to *Pc* compared to progeny from parents exposed to the lowest level of *Pst* disease; however, this difference was not statistically significant (Figure 6A and Supplementary Figure 5A). By contrast, the increased sensitivity of F1 progeny from *Pst*-exposed parents to salt showed statistically significant differences that were proportional to the levels of parental disease severity (Figure 6B and Supplementary Figure 5C). Similarly, *Hpa* susceptibility in F1 progeny from *Pc*-exposed parents (Figure 6C and Supplementary Figure 6A), as well as non-specific t-IR in F1 progeny from salt-exposed parents, showed statistically significant differences that were proportional to the level of parental stress (Figures 6E,F and Supplementary Figure 7). Thus, although the intensity of t-IR by *Pst* and *Pc* in matched environments of F1 progeny does not show a statistically significant dose effect, both responses are associated with dose-dependent costs that become evident in mismatched environments. Finally, we investigated whether the transgenerational stability of t-IR into the F2 generation is proportional to the level of parental stress in matched environments. To this end, we determined t-IR by *Pst* and *Pc* in F2 progeny in matching environments after one stress-free F1 generation. In contrast to F1 progeny (Figure 3B and Supplementary Figure 2B), F2 progeny from parents exposed to the lowest levels of *Pst* disease no longer showed t-IR against *Hpa* (Figure 3C and Supplementary Figure 2C). Furthermore, only one F2 population from parents exposed to intermediate levels of *Pst* disease had maintained a statistically significant t-IR response, whereas all F2 populations from parents exposed to the highest levels of *Pst* disease had maintained a statistically significant t-IR response (Figure 3C and Supplementary Figure 2C). F2 populations from parents exposed to low and intermediate levels of *Pc* disease all failed to show t-IR against *Pc* (Figure 4B and Supplementary Figure 3B). However, ANOVA of pooled F2 populations from similarly treated parental plants, as well as nested ANOVA with F2 population as a random variable, revealed a statistically significant effect of parental stress treatment (Supplementary Figure 3B), indicating a residual amount of t-IR in F2 populations from *Pc*-exposed parents. This is further supported by the observation that F2 populations from parents exposed to the highest degree of *Pc* disease also showed the highest level of *Pc* resistance (Figure 4B and Supplementary Figure 3B). Hence, t-IR in response to relatively high levels of disease by *Pst* or *Pc* can persist into the F2 generation, whereas t-IR elicited by low or intermediate disease levels is reverted or weakened after one stress-free F1 generation. Together, these results demonstrate that the intensity, costs, and/or transgenerational stability of t-IR have a dose-dependent relationship with parental stress intensity, which supports our hypothesis that plants use stress intensity as a weighted indicator of t-IR investment.

## Discussion

Transgenerational phenotypic plasticity offers a strategy for plants and animals to maximize fitness in variable environments and is more likely to emerge when the same type of environmental stress occurs regularly (Tricker, 2015; Proulx and Teotonio, 2017). Under such conditions, stress-exposed plants can optimize fitness either by maximizing their own immediate performance to the detriment of their progeny (“selfish parental effects”), or by modifying progeny traits to provide enhanced performance in the altered environment (Marshall and Uller, 2007). The latter strategy can take form in either a diversified bet-hedging strategy, or a more deterministic provision of specific adaptive traits, such as (transgenerational) pathogen-IR, which is tailored to the parental environment (Marshall and Uller, 2007; Crean and Marshall, 2009; Proulx and Teotonio, 2017). Not only do evolutionary models predict that transgenerational phenotypic plasticity is likely to evolve in fluctuating environments (Leimar and McNamara, 2015; Pigeault et al., 2016; Proulx and Teotonio, 2017), the model developed by Proulx and Teotonio (2017) suggests that deterministic parental effects provide increased fitness over a wider range of environmental parameters than a randomizing bet-hedging strategy. Such models assume that parental effects are specific to the eliciting stress, involve cost–benefit trade-offs, and that parents have access to reliable predictive cues.

Our study employed a full factorial design to address these predictions in relation to transgenerational responses of Arabidopsis to different (a)biotic stresses. Most previous reports have not, or only partially, addressed the specificity of t-IR, because they only tested t-IR upon a single parental stress and/or in matched environments. By contrast, the experiential design of our study (Figure 1) allowed us to examine t-IR phenotypes in progeny from parents exposed to different stresses in both matched and mismatched environments and in a reciprocal fashion. In the case of disease stress, we found strong evidence that t-IR is specific. Disease by biotrophic *Pst* bacteria mediates t-IR against taxonomically unrelated *Hpa* with a similar biotrophic lifestyle (Figures 3B,C) but fails to protect against necrotrophic *Pc* or abiotic salt stress (Figures 6A–C). Similarly, disease by *Pc* mediated t-IR against the same necrotrophic fungus (Figure 4), but not against biotrophic *Hpa* and abiotic salt stress (Figures 6C,D). While we found specificity of t-IR responses to biotic stress, other studies have provided examples of both specific and non-specific t-IR, especially in response to abiotic stress. Progeny of tobacco plants infected with tobacco mosaic virus displayed t-IR against both biotrophic and necrotrophic pathogens, as well as the genotoxic agent methyl methane sulfonate (MMS; Kathiria et al., 2010), while exposure of Arabidopsis to different heavy metals caused t-IR not only against the same heavy metals, but also against sodium chloride and MMS (Rahavi et al., 2011).

By contrast, we found no evidence for increased salt tolerance in F1 or F2 progeny from salt-exposed parents (Figure 5), even though F1 progeny from salt-exposed parents showed non-specific t-IR against biotrophic and necrotrophic pathogens (Figures 6E,F). Several previous studies have demonstrated t-IR against salinity (Boyko et al., 2010; Wibowo et al., 2016). However, the parental plants used in these studies were allowed to recover from the salt stress by transplanting them to normal soil. By contrast, the parental plants in our experiments were not transplanted and showed progressive loss of seed production and viability at increasing levels of salinity (Figures 2B,C), which indicates that they failed to recover from the stress. Accordingly, we propose that parental recovery from salt stress is essential for salt-mediated t-IR in matched environments. This hypothesis is supported by modeling, which shows that t-IR in invertebrates emerges at intermediate levels of disease stress, but not when there are more severe impacts resulting in mortality (Pigeault et al., 2016). By contrast, the pathogen-inoculated parental plants in our study were able to prevent negative impacts on reproductive fitness in our experiments (Figures 2B,C), presumablyby effective deployment of within-generation immune responses and subsequent compensatory growth during reproduction that maintained seed production. Similar compensatory responses were observed in Arabidopsis exposed to wounding, shading, or chilling stress during vegetative development (Lampei, 2019). Under these conditions, t-IR represents an ecological benefit for progeny in matched environment. One might also speculate that the observed differences t-IR effectiveness in our study reflect the nature of the eliciting stresses. Pathogen populations tend to oscillate naturally, and fluctuating environments are consistent with the evolution of adaptive parental effects (Leimar and McNamara, 2015; Proulx and Teotonio, 2017). In contrast, salinity would more likely be long term as opposed to a variable stress in natural environments, favoring genetic, rather than epigenetic adaptation.

The fact that pathogen-mediated t-IR is inducible and reversible in the absence of stress (Figures 3, 4) implies that the response is associated with costs (Vos et al., 2013; Karasov et al., 2017). The costs of defense can manifest themselves either as direct allocation costs, where investment in defense reduces resources available for growth and reproduction, or as ecological costs, when defense responses impact wider plant–environment interactions. Priming of defense has likely arisen as a mechanism to optimize cost–benefit trade-offs of induced resistance responses. In response to a primary exposure to stress, which indicates possible re-exposure in the future, plants prime defenses for more rapid and/or stronger responses (Wilkinson et al., 2019). Accordingly, naïve plants that have not been exposed to stress avoid costs completely by not deploying priming, whereas plants pre-exposed to the stress gain greater benefit from a more effective inducible defense response. This cost–benefit optimization by priming has previously been confirmed experimentally for within-generation priming (van Hulten et al., 2006), but the same rationale applies equally to between generation priming by t-IR (Karasov et al., 2017). Previous work has identified transgenerational impacts of parental stress on vegetative and reproductive development (e.g., Rahavi et al., 2011; Suter and Widmer, 2013; Groot et al., 2016), but it remains unclear to what extent these changes influence fitness. While we did not observe consistent effects on plant growth or seed set in F1 and F2 progeny from disease-exposed plants (data not shown), the reciprocal design of our experiments reveals ecological costs arising from increased susceptibility to other stresses (Figure 6). Antagonism between plant defense pathways against biotrophic pathogens, necrotrophic pathogens, and abiotic stress is well documented (Koornneef and Pieterse, 2008; Pieterse et al., 2012), and transgenerational persistence of these effects has been reported previously (Luna et al., 2012; Singh et al., 2017). Accordingly, we propose that negative cross-talk between defense pathways imposes a major ecological cost on t-IR responses to pathogens.

Central to the provision of adaptive transgenerational traits is the ability to make accurate and reliable predictions about future progeny environments. While this aspect has been emphasized in both evolutionary theory and modeling of parental effects (Burgess and Marshall, 2014; Leimar and McNamara, 2015), it has rarely been addressed experimentally. Rahavi and Kovalchuk (2013) tested the effect on homologous recombination frequency (HRF) and leaf size, of varying the timing and duration of exposure to several different abiotic stresses in Arabidopsis plants and their progeny. Overall, there was little evidence for dose-dependency, except that recombination events occurred earlier during the development of parent plants as doses of UV-C irradiation increased. HRF in progeny plants was not related to the duration of stress experienced by parents and t-IR against stress was not tested in these experiments. Other plant studies addressing this concept applied the same stress repeatedly over multiple generations. For instance, analysis of plants exposed to heavy metal stress for up to five successive generations indicated increasing tolerance as the number of previous generations experiencing stress increased (Rahavi et al., 2011). In one of the most comprehensive studies of this type, Groot et al. (2016) found complex interactions between parental (P), grandparental (GP), and great-grandparental (GGP) salt stress in Arabidopsis. When the stress was applied to only one generation, P effects were typically stronger than GP and GGP effects. For treatments over multiple generations, the impacts of GP and GGP stress were additive to P treatments for some traits, but antagonistic for others. In our study, varying levels of three different stresses were applied within one generation, providing a straightforward design to assess whether parents can distinguish stress severities and adjust the transgenerational response accordingly. Our pathogen treatments resulted in dose-dependent impacts on relative growth rate during the treatment period (Figure 2A), indicating that Arabidopsis perceives these stresses in a dose-dependent manner. Furthermore, analysis of the transgenerational stability of t-IR provided evidence for a dose-dependent relationship with parental disease severity. Although F1 populations from both *Pst-* and *Pc-*exposed parents expressed t-IR to statistically similar levels across stress levels (Figures 3A,B, 4A), these t-IR responses only persisted over a stress-free generation when elicited by the highest stress levels (Figures 3C, 4B). Furthermore, in mismatched environments, there was a dose-dependent effect on the costs t-IR by pathogens: both salt sensitivity of F1 progeny from *Pst*-infected parents and *Hpa* susceptibility of F1 progeny from *Pc*-infected parents correlated with the severity of parental disease stress treatment (Figures 6B,C). Overall, these results support our hypothesis that plants perceive disease severity as a predictive cue to adjust t-IR investment.

Collectively, our study demonstrates that parental investment in t-IR by pathogen stress provides benefits in matched environments and costs in mismatched environments. This stress-specific t-IR is dependent on the intensity of the stress experienced by the parents, which holds predictive value for future progeny environments. Accordingly, our findings are consistent with the evolutionary prediction that t-IR by disease stress from pathogens is an adaptive trait. In laboratory experiments, true measures of evolutionary fitness are difficult to estimate, since plants are grown in controlled environments in the absence of competition from non-stressed plants and other external factors, such as seasonal changes in temperature and photoperiod that can influence reproductive development. In one of the most convincing cases of adaptive parental effects in plants, Galloway and Etterson (2007) used field-based studies to demonstrate adaptive transgenerational plasticity in response to the light environment. It will now be of interest to extend our laboratory experiments by undertaking ecological field studies to seek support for the concept of t-IR as an adaptive transgenerational effect in nature.

## Data Availability Statement

The raw data supporting the conclusions of this article will be made available by the authors, without undue reservation.

## Author Contributions

JT and MR conceived the project. JT and AL designed and supervised the experiments. AL, DP-P, and LF performed bioassays. JT performed statistical analyses. MR, AL, and JT wrote the manuscript. All authors reviewed and approved the final manuscript.

## Conflict of Interest

The authors declare that the research was conducted in the absence of any commercial or financial relationships that could be construed as a potential conflict of interest.

## Footnotes

1 https://rstudio.com/

2 https://www.r-project.org/

## Supplementary Material

Supplementary Figure 1 Impacts of (a)biotic stresses on seed viability from individual plants. See the legend of Figure 2C for details. Boxplots show the interquartile range (IQR; box) ± 1.5xIQR (whiskers), including median (horizontal line) and replication units (dots). Data represent germination percentages of F1 seed batches from *Pst*-exposed parents [green **(A)**; *n* = 3–4], F1 seeds from *Pc*-exposed parents [blue **(C)**; *n* = 3–4], F1 seeds from salt-exposed parents [orange **(C)**; *n* = 8–22], and F2 seeds from salt-exposed parents after one stress-free F1 generation [orange **(D)**; *n* = 4–5). *P*-values indicate statistical significance of parent treatment by Welch ANOVA of pooled populations from similarly treated parent plants (F1) or a common parental ancestor (F2), as well as nested ANOVA with individual F1/F2 population as random factor. Different letters indicate statistically significant differences between pooled populations (Welch ANOVA + Games-Howell *post hoc* test; α = 0.05; NS, no statistically significant differences).

Supplementary Figure 2 t-IR in individual F1 and F2 populations from *Pst*-treated parents in matched environments. **(A)** *Pst*-mediated t-IR against *Pst* in F1 plants at 3 dpi. See the legend of Figure 3A for details. Boxplots show the interquartile range (IQR; box) ± 1.5xIQR (whiskers), including median (horizontal line) and replication units (dots). Data represent ^10^log-transformed bacterial titers (log cfu cm^–2^; *n* = 10–12) in leaves of single plants within individual F1 populations. *P*-values indicate statistical significance of parent treatment by Welch ANOVA of pooled F1 populations from similarly treated parents, and nested ANOVA with individual F1 population as random factor, respectively. Different letters indicate statistically significant differences between pooled F1 populations from similarly treated parents (Welch ANOVA + Games-Howell *post hoc* test; α = 0.05). **(A)** *Pst*-mediated t-IR against *Hpa* in F1 plants at 6 dpi. See the legend of Figure 3A for details. Stacked bars show leaf frequency distributions across *Hpa* resistance classes within individual F1 populations (*n* = 70–250). *P*-value indicates statistical significance of the parent treatment (Fisher’s exact test). Different letters indicate statistically significant differences between pooled F1 populations from similarly treated parents (pairwise Fisher’s exact tests + Bonferroni FDR, α = 0.05). **(C)** *Pst*-mediated t-IR against *Hpa* in F2 plants at 6 dpi after one stress-free F1 generation. See the legend of Figure 3A for details. Stacked bars show leaf frequency distributions across *Hpa* resistance classes within individual F2 populations (*n* = 55–100). *P*-value indicates statistical significance of parent treatment (Fisher’s exact tests). Different letters indicate statistically significant differences between pooled F2 populations from a common parental ancestor (pairwise Fisher’s exact tests + Bonferroni FDR; α = 0.05).

Supplementary Figure 3 t-IR in individual F1 and F2 populations from *Pc*-exposed parents in matched environments. Data represent lesion diameters (mm) of plants within individual populations at 15 dpi. See the legend of Figure 4 for details. Boxplots show the interquartile range (IQR; box) ± 1.5xIQR (whiskers), including median (horizontal line) and replication units (dots). **(A)** *Pc*-mediated t-IR against *Pc* in F1 plants (*n* = 10). *P*-values indicate statistical significance of parent treatment by Welch ANOVA of pooled F1 populations from similarly treated parents, and nested ANOVA with F1 population as random factor, respectively. Different letters indicate statistically significant differences between pooled F1 populations from similarly treated parents (Welch ANOVA + Games-Howell *post hoc* test; α = 0.05). **(B)** *Pc*-mediated t-IR against *Pc* in F2 plants (*n* = 5). *P*-values indicate statistical significance of parent treatment by ANOVA of pooled F2 populations from a common parental ancestor, and nested ANOVA with F2 population as random factor, respectively. Different letters indicate statistically significant differences between pooled F2 populations from a common parental ancestor (ANOVA + Tukey’s *post hoc* test; α = 0.05).

Supplementary Figure 4 Transgenerational effects of soil salinity on root growth and salt tolerance in individual F1 and F2 populations and matched environments. All boxplots show the interquartile range (IQR; box) ± 1.5xIQR (whiskers), including median (horizontal line) and replication units (dots). **(A)** Root growth of F1 plants at 0, 50, and 100 mM NaCl. Data represent root growth values (cm) of plants within individual F1 populations over a 5-day period (*n* = 15). *P*-values indicate statistical significance of parent treatment, F1 treatment, and interaction by two-way ANOVA of pooled F1 populations from similarly treated parents, and nested two-way ANOVA with F1 population as random factor, respectively. For each NaCl concentration, different letters indicate statistically significant differences between pooled F1 populations from similarly treated parents (ANOVA + Tukey’s *post hoc* test; α = 0.05; NS, no significant differences). **(B)** Root growth of F2 plants at 0, 50, and 100 mM NaCl after one stress-free F1 generation. Data represent root growth values (cm) of plants within individual F2 populations over a 5-day period (*n* = 4–5). *P*-values indicate statistical significance of parent treatment, F2 treatment, and interaction by two-way ANOVA of pooled F2 populations from a common parental ancestor, and nested two-way ANOVA with F2 population as random factor, respectively. NS, no statistically significant differences between pooled F2 populations from a common parental ancestor (ANOVA + Tukey’s *post hoc* test; α = 0.05). **(C)** Tolerance of F1 plants to 50 and 100 mM NaCl. Tolerance was quantified by root growth reduction (%) relative to the mean root growth at 0 mM NaCl of the corresponding F1 population **(A)**. Data represent growth reduction percentages of single plants within individual F1 populations (*n* = 15). *P*-values indicate statistical significance of parent treatment, F1 treatment, and interaction by two-way ANOVA of pooled F1 populations from similarly treated parents, and nested two-way ANOVA with F1 population as random factor, respectively. NS, no statistically significant differences between pooled F1 populations from similarly treated parents (ANOVA + Tukey’s *post hoc* test; α = 0.05). **(D)** Tolerance of F2 plants to 50 and 100 mM NaCl after one stress-free F1 generation. Tolerance was quantified by root growth reduction relative to the mean root growth at 0 mM NaCl of the corresponding F2 population **(B)**. Data represent growth reduction percentages of single plants within individual F2 populations (*n* = 4–5). *P*-values indicate statistical significance of parent treatment, F2 treatment, and interaction by two-way ANOVA of pooled F2 populations from a common parental ancestor, and nested two-way ANOVA with F2 population as random factor, respectively. NS, no statistically significant differences between pooled F2 populations from a common parental ancestor (ANOVA + Tukey’s *post hoc* test; α = 0.05).

Supplementary Figure 5 Costs of *Pst*-mediated t-IR in individual F1 populations and mismatched environments. All boxplots show the interquartile range (IQR; box) ± 1.5xIQR (whiskers), including median (horizontal line) and replication units (dots). See the legends of Figures 6A,B for details. **(A)** *Pc* resistance of F1 plants from mock- and *Pst*-treated parents 6 dpi. Data represent lesion diameters (mm) of plants within individual F1 populations (*n* = 38–40). *P*-values indicate statistical significance of parent treatment by ANOVA of pooled F1 populations from similarly treated parents, and nested ANOVA with individual F1 population as random factor, respectively. Different letters indicate statistically significant differences between pooled F1 populations from similarly treated parents (ANOVA + Tukey’s *post hoc* test; α = 0.05). **(B)** Root growth of F1 plants from mock- and *Pst*-treated parents at 0, 50, and 100 mM NaCl. Data represent root growth values (cm) of plants within individual F1 populations over a 5-day period (*n* = 10). *P*-values indicate statistical significance of parent treatment, F1 treatment and interaction by two-way ANOVA of pooled F1 populations from similarly treated parental plants, and nested 2-way ANOVA with F1 population as random factor, respectively. For each NaCl concentration, different letters indicate statistically significant differences between pooled F1 populations from similarly treated parents (ANOVA + Tukey’s *post hoc* test; α = 0.05; NS, no statistically significant differences). **(C)** Tolerance of F1 plants from mock- and *Pst*-treated parents to 50 and 100 mM NaCl. Tolerance was quantified by root growth reduction (%) relative to the mean root growth value at 0 mM NaCl of the corresponding F1 population **(B)**. Data represent root growth reduction percentages of single plants within individual F1 populations (*n* = 10). *P*-values indicate statistical significance of parent treatment, F1 treatment, and interaction by two-way ANOVA of pooled F1 populations from similarly treated parents, and by nested two-way ANOVA with F1 population as random factor, respectively. For each NaCl concentration, different letters indicate statistically significant differences between pooled F1 populations from similarly treated parents (ANOVA + Tukey’s *post hoc* test; α = 0.05; NS, no significant differences).

Supplementary Figure 6 Costs of *Pc*-mediated t-IR in individual F1 populations and mismatched environments. See the legends of Figures 6C,D for details. **(A)** *Hpa* resistance in F1 plants from *Pc*-exposed parents. Stacked bars show leaf frequency distributions across *Hpa* resistance classes within F1 populations from similarly treated parents (*n* = 80–130). *P*-value indicates statistical significance of parental treatment (Fisher’s exact test). Different letters indicate statistically significant differences between pooled F1 populations from similarly treated parents (Pairwise Fisher’s exact tests + Bonferroni FDR; α = 0.05). **(B)** Root growth of F1 plants from mock- and *Pc*-treated parents at 0, 50, and 100 mM NaCl. Boxplots show the interquartile range (IQR; box) ± 1.5xIQR (whiskers), including median (horizontal line) and replication units (dots). Data represent root growth values (cm) of single plants within individual F1 populations over a 5-day period (*n* = 10). *P*-values indicate statistical significance of parent treatment, F1 treatment, and interaction by two-way ANOVA of pooled F1 populations from similarly treated parents, and nested two-way ANOVA with F1 population as random factor, respectively. NS, no statistically significant differences between pooled F1 populations from similarly treated parents (ANOVA + Tukey’s *post hoc* test; α = 0.05). **(C)** Tolerance of F1 plants from mock- and *Pc*-treated parents to 50 and 100 mM NaCl. Tolerance was quantified by root growth reduction (%) relative to the mean root growth value at 0 mM NaCl of the corresponding F1 population **(B)**. Boxplots show the interquartile range (IQR; box) ± 1.5xIQR (whiskers), including median (horizontal line) and replication units (dots). Data represent root growth reduction percentages of single plants within individual F1 populations. *P*-values indicate statistical significance of parent treatment, F1 treatment, and interaction by two-way ANOVA of pooled F1 populations from similarly treated parents, and nested two-way ANOVA with F1 population as random factor, respectively. NS, no statistically significant differences between pooled F1 populations from similarly treated parents (ANOVA + Tukey’s *post hoc* test; α = 0.05).

Supplementary Figure 7 Non-specific t-IR by soil salinity against *Pst* and *Pc* in individual F1 populations and mismatched environments. See the legends of Figures 6E,F for details. **(A)** Non-specific salt-mediated t-IR against *Hpa* in F1 plants. Stacked bars show leaf frequency distributions across *Hpa* resistance classes within individual F1 populations (*n* = 100–225). *P*-value indicates statistical significance of parental treatment (Fisher’s exact test). Different letters indicate statistically significant differences between pooled F1 populations from similarly treated parental plants (pairwise Fisher’s exact tests + Bonferroni FDR; α = 0.05). **(B)** Non-specific salt-mediated t-IR against *Pc* in F1 plants. Boxplots show the interquartile range (IQR; box) ± 1.5xIQR (whiskers), including median (horizontal line) and replication units (dots). Data represent lesion diameters (mm) of plants within individual F1 populations (*n* = 15). *P*-values on the right indicate statistical significance of parent treatment by ANOVA of pooled F1 populations from similarly treated parental plants, and nested ANOVA with individual F1 population as random factor, respectively. Different letters indicate statistically significant differences between pooled F1 populations from similarly treated parents (ANOVA + Tukey’s *post hoc* test; α = 0.05).

Supplementary Table 1 Collection of Arabidopsis F1 and F2 populations (Col-0) that in the parental generation had been subjected to varying stress intensities by *Pst*, *Pc*, or soil salinity.

Supplementary Table 2 Number of independent t-IR experiments with similar results in matched environments. ^∗^3 and 2 repeats in F1 and F2 progeny, respectively, were only testing in response to the highest stress level.

Supplementary Table 3 Number of independent t-IR experiments with similar results in mismatched environments.
