# Supplementary material for: Costs and Benefits of Transgenerational Induced Resistance in Arabidopsis
Source: Front Plant Sci. 2021 Feb 26;12:644999. doi: 10.3389/fpls.2021.644999 (PMC7952753; doi:10.3389/fpls.2021.644999)
Supplement: Supplementary Figure 1 — Impacts of (a)biotic stresses on seed viability from individual plants. See the legend of Figure 2C for details. Boxplots show the interquartile range (IQR; box) ± 1.5xIQR (whiskers), including median (horizontal line) and replication units (dots). Data represent germination percentages of F1 seed batches from Pst-exposed parents [green (A); n = 3–4], F1 seeds from Pc-exposed parents [blue (C); n = 3–4], F1 seeds from salt-exposed parents [orange (C); n = 8–22], and F2 seeds from salt-exposed parents after one stress-free F1 generation [orange (D); n = 4–5). P-values indicate statistical significance of parent treatment by Welch ANOVA of pooled populations from similarly treated parent plants (F1) or a common parental ancestor (F2), as well as nested ANOVA with individual F1/F2 population as random factor. Different letters indicate statistically significant differences between pooled populations (Welch ANOVA + Games-Howell post hoc test; α = 0.05; NS, no statistically significant differences). [file Data_Sheet_1.pdf]

Supplementary Figures

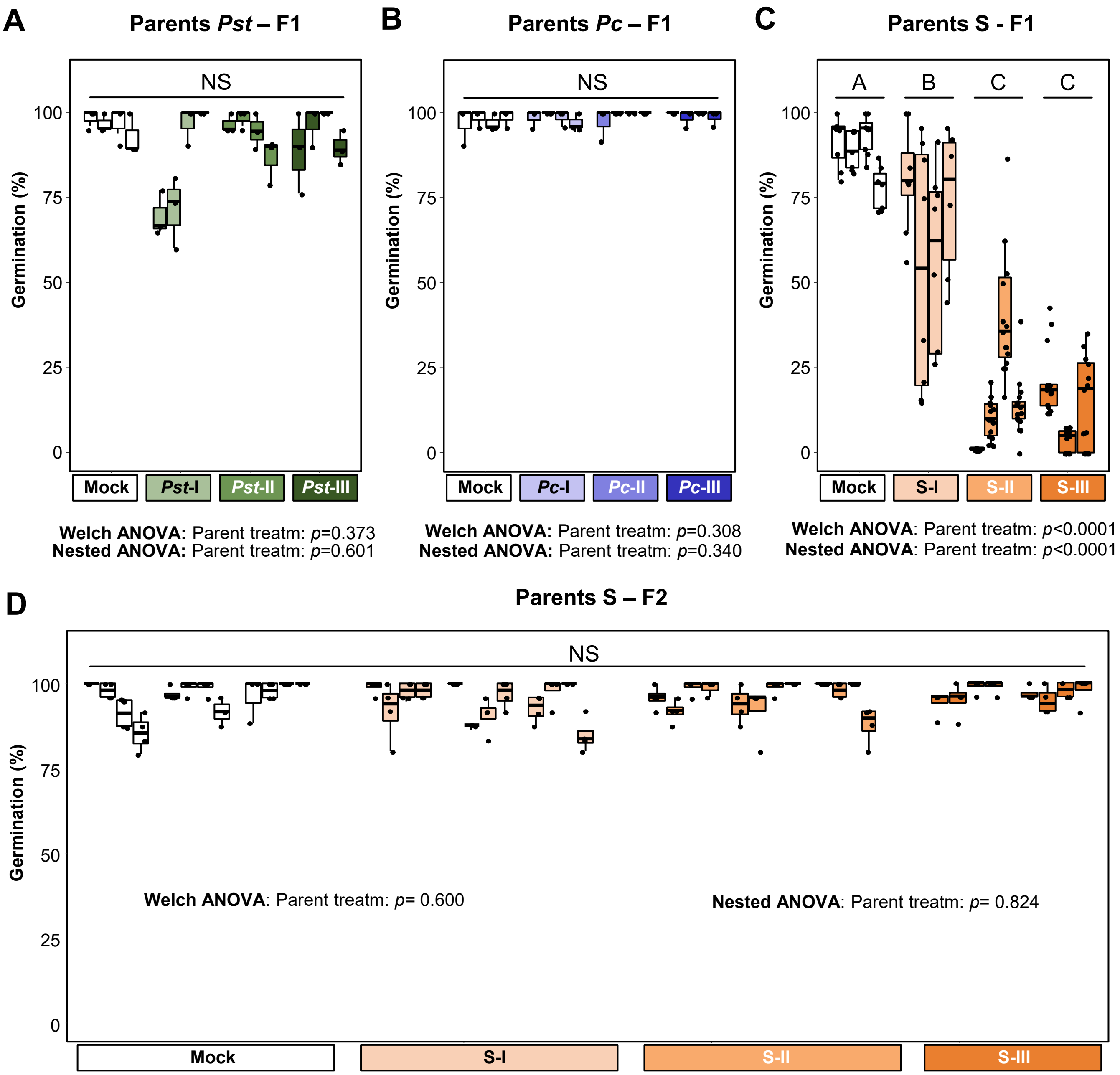

Figure S1: Impacts of (a)biotic stresses on seed viability from individual plants. See legend of Fig. 2C for details. Boxplots show the interquartile range (IQR; box) ± 1.5xIQR (whiskers), including median (horizonal line) and replication units (dots). Data represent germination percentages of F1 seed batches from *Pst*-exposed parents (green, **(A)**; n=3-4), F1 seeds from *Pc*-exposed parents (blue, **(B)**; n=3-4), F1 seeds from salt-exposed parents (orange, **(C)**; n=8-22) and F2 seeds from salt-exposed parents after one stress-free F1 generation (orange, **(D)**; n=4-5). *P*-values indicate statistical significance of parent treatment by Welch ANOVA of pooled populations from similarly treated parent plants (F1) or a common parental ancestor (F2), as well as nested ANOVA with individual F1/F2 population as random factor. Different letters indicate statistically significant differences between pooled populations (Welch ANOVA + Games-Howell post-hoc test; α=0.05; NS: no statistically significant differences).

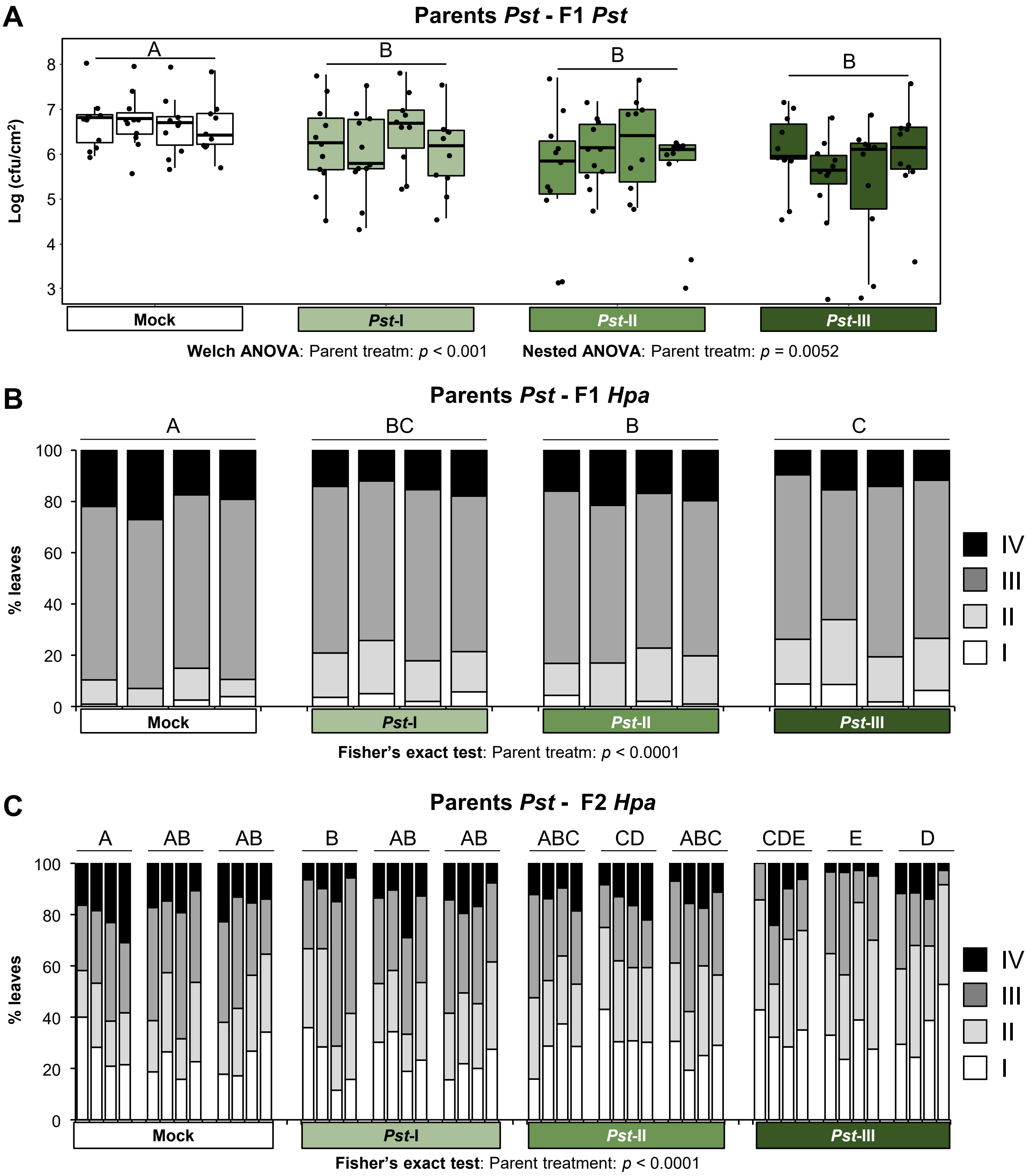

Figure S2: t-IR in individual F1 and F2 populations from *Pst*-treated parents in matched environments. (A) *Pst*-mediated t-IR against *Pst* in F1 plants at 3 dpi. See legend of Fig. 3A for details. Boxplots show the interquartile range (IQR; box) ± 1.5xIQR (whiskers), including median (horizonal line) and replication units (dots). Data represent ^{10}Log-transformed bacterial titres (Log cfu cm^{-2}; n=10-12) in leaves of single plants within individual F1 populations. *P*-values indicate statistical significance of parent treatment by Welch ANOVA of pooled F1 populations from similarly treated parents, and nested ANOVA with individual F1 population as random factor, respectively. Different letters indicate statistically significant differences between pooled F1 populations from similarly treated parents (Welch ANOVA + Games-Howell post-hoc test; α=0.05). **(B)** *Pst*-mediated t-IR against *Hpa* in F1 plants at 6 dpi. See legend of Fig. 3A for details. Stacked bars show leaf frequency distributions across *Hpa* resistance classes within individual F1 populations (n=70-250). *P*-value indicates statistical significance of the parent treatment (Fisher's exact test). Different letters indicate statistically significant differences between pooled F1 populations from similarly treated parents (pairwise Fisher's exact tests + Bonferroni FDR, α=0.05). **(C)** *Pst*-mediated t-IR against *Hpa* in F2 plants at 6 dpi after one stress-free F1 generation. See legend of Fig. 3A for details. Stacked bars show leaf frequency distributions across *Hpa* resistance classes within individual F2 populations (n=55-100). *P*-value indicates statistical significance of parent treatment (Fisher's exact tests). Different letters indicate statistically significant differences between pooled F2 populations from a common parental ancestor (pairwise Fisher's exact tests + Bonferroni FDR; α=0.05).

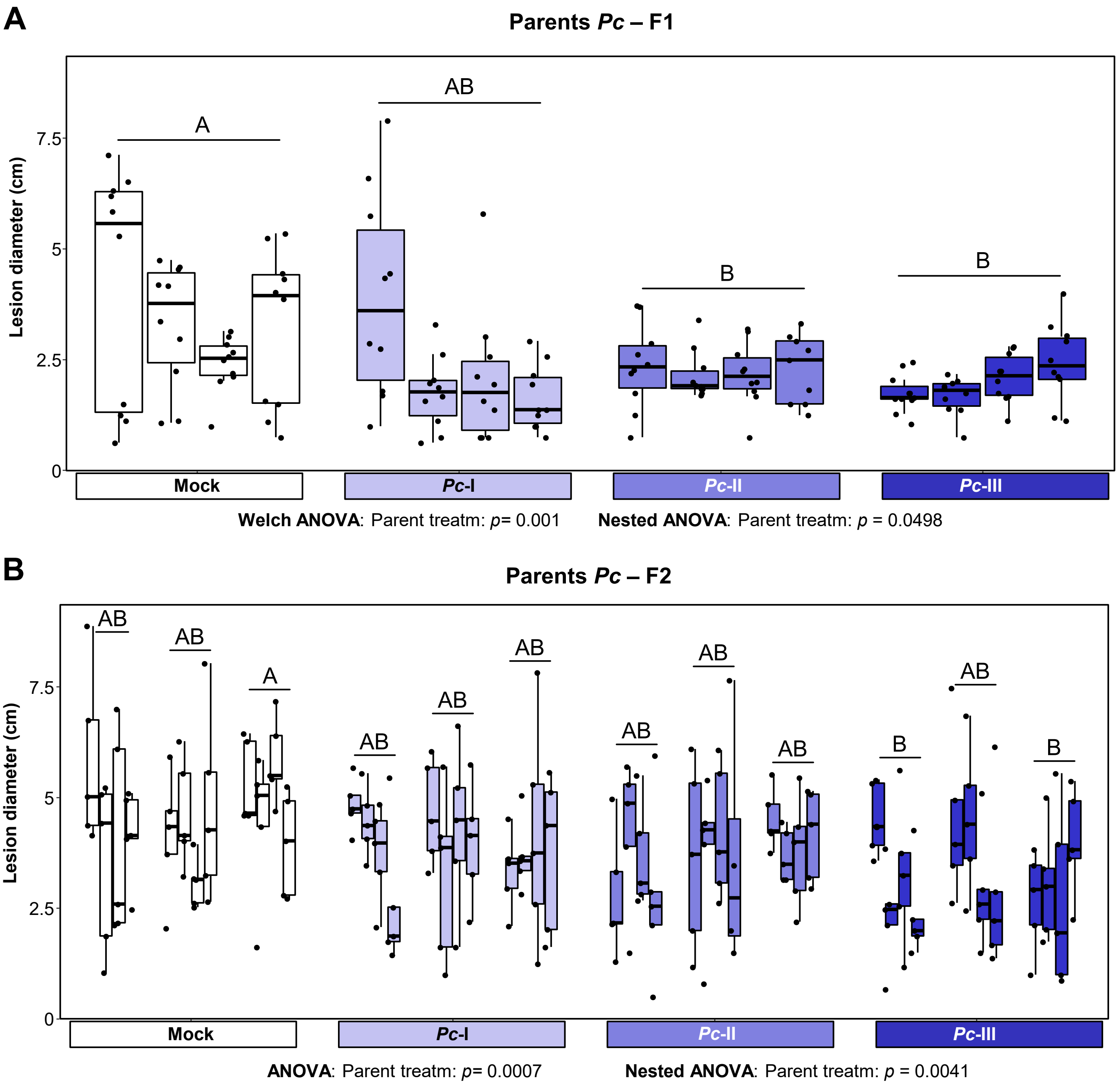

Figure S3. t-IR in individual F1 and F2 populations from *Pc*-exposed parents in matched environments. Data represent lesion diameters (mm) of plants within individual populations at 15 dpi. See legend of Fig. 4 for details. Boxplots show the interquartile range (IQR; box) ± 1.5xIQR (whiskers), including median (horizonal line) and replication units (dots). **(A)** *Pc*-mediated t-IR against *Pc* in F1 plants (n=10). *P*-values indicate statistical significance of parent treatment by Welch ANOVA of pooled F1 populations from similarly treated parents, and nested ANOVA with F1 population as random factor, respectively. Different letters indicate statistically significant differences between pooled F1 populations from similarly treated parents (Welch ANOVA + Games-Howell post-hoc test; α=0.05). **(B)** *Pc*-mediated t-IR against *Pc* in F2 plants (n=5). *P*-values indicate statistical significance of parent treatment by ANOVA of pooled F2 populations from a common parental ancestor, and nested ANOVA with F2 population as random factor, respectively. Different letters indicate statistically significant differences between pooled F2 populations from a common parental ancestor (ANOVA + Tukey post-hoc test; α=0.05).

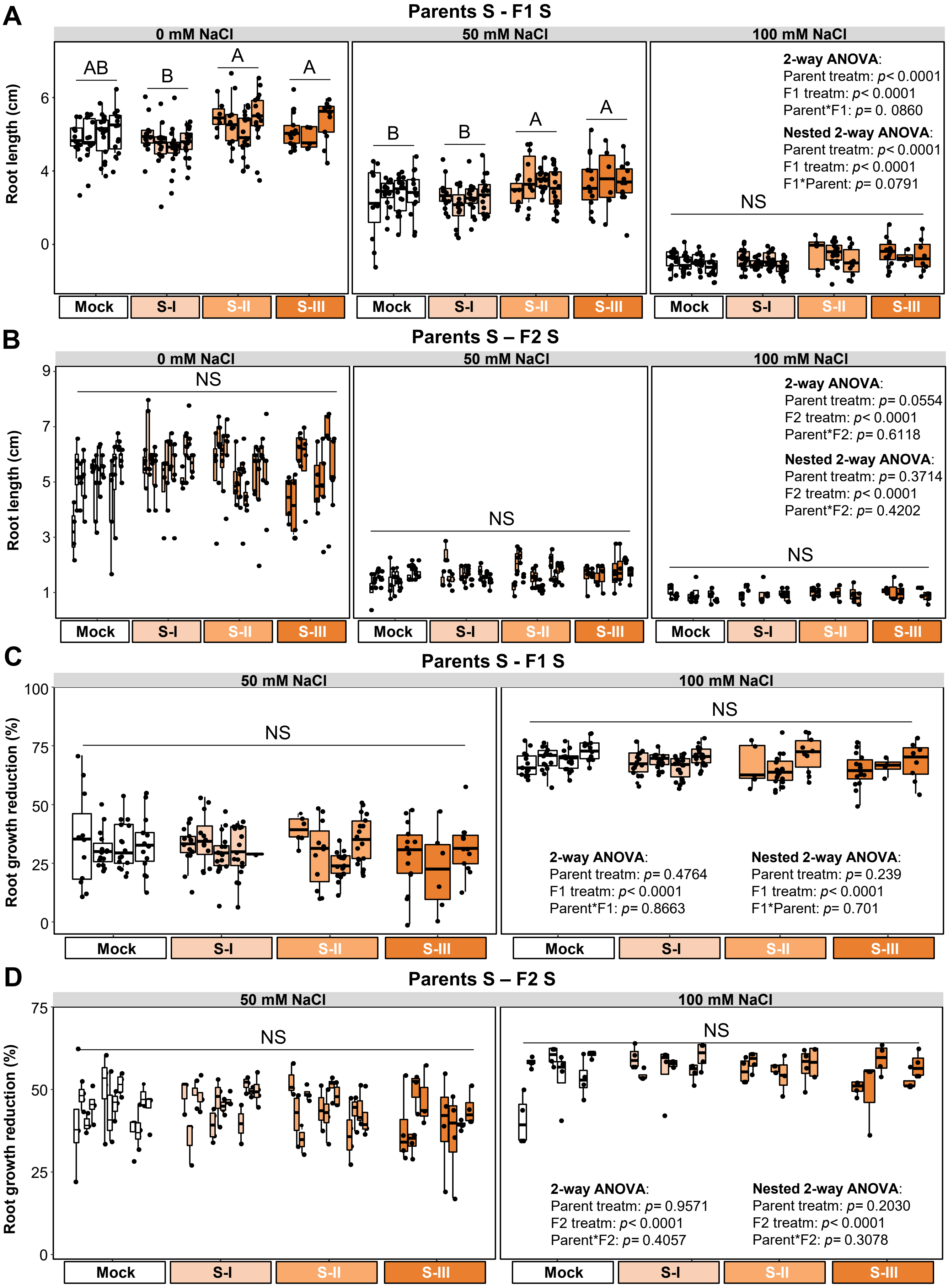

Figure S4. Transgenerational effects of soil salinity on root growth and salt tolerance in individual F1 and F2 populations and matched environments. All boxplots show the interquartile range (IQR; box) ± 1.5xIQR (whiskers), including median (horizonal line) and replication units (dots). **(A)** Root growth of F1 plants at 0, 50 and 100 mM NaCl. Data represent root growth values (cm) of plants within individual F1 populations over a 5-day period (n=15). *P*-values indicate statistical significance of parent treatment, F1 treatment and interaction by 2-way ANOVA of pooled F1 populations from similarly treated parents, and nested 2-way ANOVA with F1 population as random factor, respectively. For each NaCl concentration, different letters indicate statistically significant differences between pooled F1 populations from similarly treated parents (ANOVA + Tukey post-hoc test; α=0.05; NS: no significant differences). **(B)** Root growth of F2 plants at 0, 50 and 100 mM NaCl after one stress-free F1 generation. Data represent root growth values (cm) of plants within individual F2 populations over a 5-day period (n=4-5). *P*-values indicate statistical significance of parent treatment, F2 treatment and interaction by 2-way ANOVA of pooled F2 populations from a common parental ancestor, and nested 2-way ANOVA with F2 population as random factor, respectively. NS: no statistically significant differences between pooled F2 populations from a common parental ancestor (ANOVA + Tukey post-hoc test; α=0.05). **(C)** Tolerance of F1 plants to 50 and 100 mM NaCl. Tolerance was quantified by root growth reduction (%) relative to the mean root growth at 0 mM NaCl of the corresponding F1 population (Fig. S4A). Data represent growth reduction percentages of single plants within individual F1 populations (n=15). *P*-values indicate statistical significance of parent treatment, F1 treatment and interaction by 2-way ANOVA of pooled F1 populations from similarly treated parents, and nested 2-way ANOVA with F1 population as random factor, respectively. NS: no statistically significant differences between pooled F1 populations from similarly treated parents (ANOVA + Tukey post-hoc test; α=0.05). **(D)** Tolerance of F2 plants to 50 and 100 mM NaCl after one stress-free F1 generation. Tolerance was quantified by root growth reduction relative to the mean root growth at 0 mM NaCl of the corresponding F2 population (Fig. S4B). Data represent growth reduction percentages of single plants within individual F2 populations (n=4-5). *P*-values indicate statistical significance of parent treatment, F2 treatment and interaction by 2-way ANOVA of pooled F2 populations from a common parental ancestor, and nested 2-way ANOVA with F2 population as random factor, respectively. NS: no statistically significant differences between pooled F2 populations from a common parental ancestor (ANOVA + Tukey post-hoc test; α=0.05).

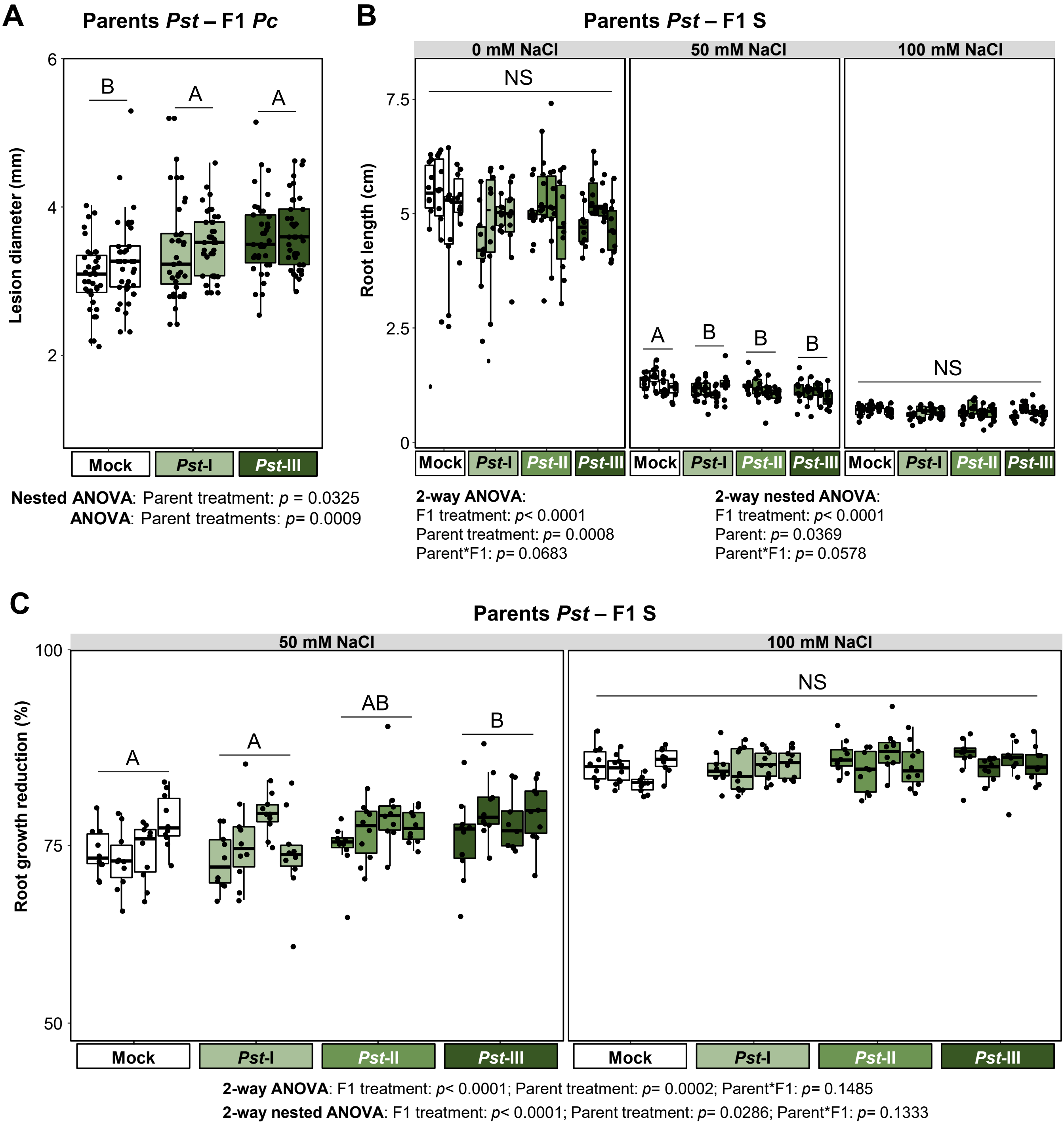

Figure S5. Costs of *Pst*-mediated t-IR in individual F1 populations and mismatched environments. All boxplots show the interquartile range (IQR; box) ± 1.5xIQR (whiskers), including median (horizonal line) and replication units (dots). See legends to Fig. 6A-B for details. **(A)** *Pc* resistance of F1 plants from mock- and *Pst*-treated parents 6 dpi. Data represent lesion diameters (mm) of plants within individual F1 populations (n=38-40). *P*-values indicate statistical significance of parent treatment by ANOVA of pooled F1 populations from similarly treated parents, and nested ANOVA with individual F1 population as random factor, respectively. Different letters indicate statistically significant differences between pooled F1 populations from similarly treated parents (ANOVA + Tukey post-hoc test; α=0.05). **(B)** Root growth of F1 plants from mock- and *Pst*-treated parents at 0, 50 and 100 mM NaCl. Data represent root growth values (cm) of plants within individual F1 populations over a 5-day period (n=10). *P*-values indicate statistical significance of parent treatment, F1 treatment and interaction by 2-way ANOVA of pooled F1 populations from similarly treated parental plants, and nested 2-way ANOVA with F1 population as random factor, respectively. For each NaCl concentration, different letters indicate statistically significant differences between pooled F1 populations from similarly treated parents (ANOVA + Tukey post-hoc test; α=0.05; NS: no statistically significant differences). **(C)** Tolerance of F1 plants from mock- and *Pst*-treated parents to 50 and 100 mM NaCl. Tolerance was quantified by root growth reduction (%) relative to the mean root growth value at 0 mM NaCl of the corresponding F1 population (Fig. S5B). Data represent root growth reduction percentages of single plants within individual F1 populations (n=10). *P*-values indicate statistical significance of parent treatment, F1 treatment and interaction by 2-way ANOVA of pooled F1 populations from similarly treated parents, and by nested 2-way ANOVA with F1 population as random factor, respectively. For each NaCl concentration, different letters indicate statistically significant differences between pooled F1 populations from similarly treated parents (ANOVA + Tukey post-hoc test; α=0.05; NS: no significant differences).

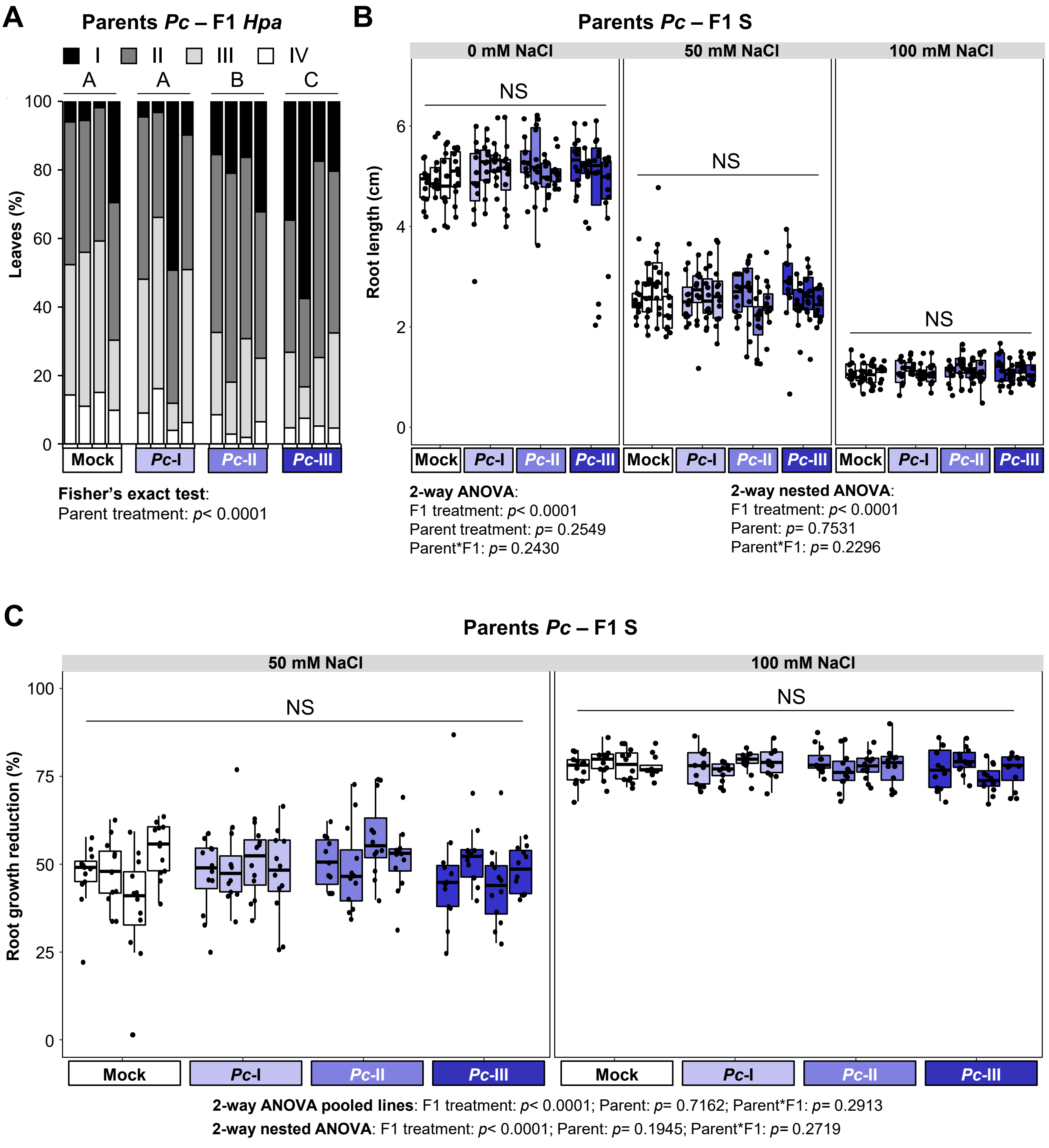

Figure S6. Costs of *Pc*-mediated t-IR in individual F1 populations and mismatched environments. See legends of Fig. 6C-D for details. **(A)** *Hpa* resistance in F1 plants from *Pc*-exposed parents. Stacked bars show leaf frequency distributions across *Hpa* resistance classes within F1 populations from similarly treated parents (n=80-130). *P*-value indicates statistical significance of parental treatment (Fisher's exact test). Different letters indicate statistically significant differences between pooled F1 populations from similarly treated parents (Pairwise Fisher's exact tests + Bonferroni FDR; α=0.05). **(B)** Root growth of F1 plants from mock- and *Pc*-treated parents at 0, 50 and 100 mM NaCl. Boxplots show the interquartile range (IQR; box) ± 1.5xIQR (whiskers), including median (horizonal line) and replication units (dots). Data represent root growth values (cm) of single plants within individual F1 populations over a 5-day period (n=10). *P*-values indicate statistical significance of parent treatment, F1 treatment and interaction by 2-way ANOVA of pooled F1 populations from similarly treated parents, and nested 2-way ANOVA with F1 population as random factor, respectively. NS: no statistically significant differences between pooled F1 populations from similarly treated parents (ANOVA + Tukey post-hoc test; α=0.05). **(C)** Tolerance of F1 plants from mock- and *Pc*-treated parents to 50 and 100 mM NaCl. Tolerance was quantified by root growth reduction (%) relative to the mean root growth value at 0 mM NaCl of the corresponding F1 population (Fig. S6b). Boxplots show the interquartile range (IQR; box) ± 1.5xIQR (whiskers), including median (horizonal line) and replication units (dots). Data represent root growth reduction percentages of single plants within individual F1 populations. *P*-values indicate statistical significance of parent treatment, F1 treatment and interaction by 2-way ANOVA of pooled F1 populations from similarly treated parents, and nested 2-way ANOVA with F1 population as random factor, respectively. NS: no statistically significant differences between pooled F1 populations from similarly treated parents (ANOVA + Tukey post-hoc test; α=0.05).

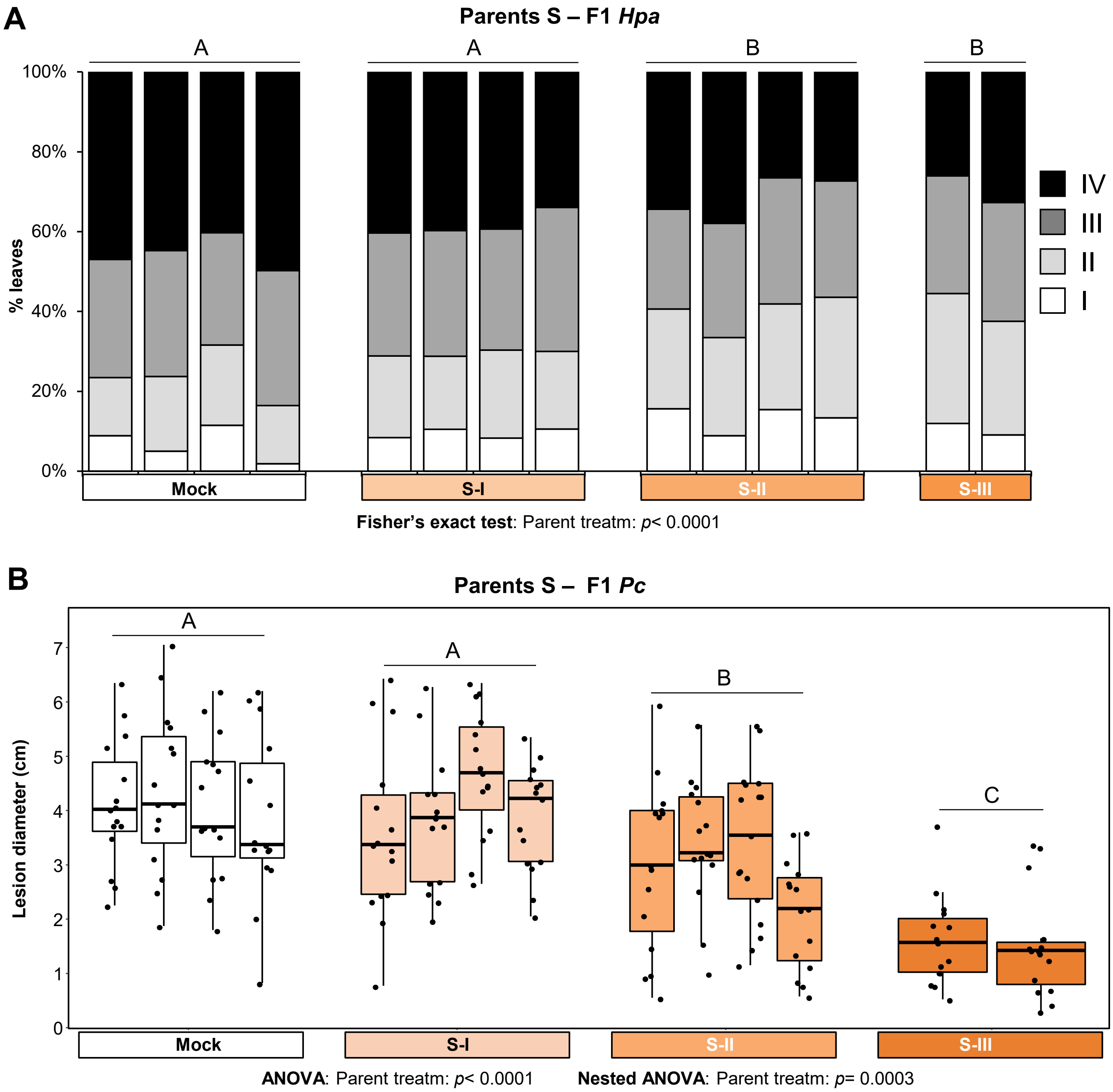

Figure S7. Non-specific t-IR by soil salinity against *Pst* and *Pc* in individual F1 populations and mismatched environments. See legend of Fig. **6E-F** for details. **(A)** Non-specific salt-mediated t-IR against *Hpa* in F1 plants. Stacked bars show leaf frequency distributions across *Hpa* resistance classes within individual F1 populations (n=100-225). *P*-value indicates statistical significance of parental treatment (Fisher's exact test). Different letters indicate statistically significant differences between pooled F1 populations from similarly treated parental plants (pairwise Fisher's exact tests + Bonferroni FDR; α=0.05). **(B)** Non-specific salt-mediated t-IR against *Pc* in F1 plants. Boxplots show the interquartile range (IQR; box) ± 1.5xIQR (whiskers), including median (horizonal line) and replication units (dots). Data represent lesion diameters (mm) of plants within individual F1 populations (n=15). *P*-values on the right indicate statistical significance of parent treatment by ANOVA of pooled F1 populations from similarly treated parental plants, and nested ANOVA with individual F1 population as random factor, respectively. Different letters indicate statistically significant differences between pooled F1 populations from similarly treated parents (ANOVA + Tukey post-hoc test; α=0.05).
