# Supplementary material for: Costs and Benefits of Transgenerational Induced Resistance in Arabidopsis
Source: Front Plant Sci. 2021 Feb 26;12:644999. doi: 10.3389/fpls.2021.644999 (PMC7952753; doi:10.3389/fpls.2021.644999)
Supplement: Supplementary Table 1 — Collection of Arabidopsis F1 and F2 populations (Col-0) that in the parental generation had been subjected to varying stress intensities by Pst, Pc, or soil salinity. [file Data_Sheet_3.pdf]

Supplementary Tables

Table S1. Collection of Arabidopsis F1 and F2 populations (Col-0) that in the parental generation had been subjected to varying stress intensities by *Pst*, *Pc* or soil salinity.

Parental treatment	Generation	No. of lines	Code
Pst-Mock	F1	4 lines	*Pst*-0, 1-4
Pst-Low	F1	4 lines	*Pst*-I, 1-4
Pst-Medium	F1	4 lines	*Pst*-II, 1-4
Pst-High	F1	4 lines	*Pst*-III, 1-4
Pst-Mock	F2	12 lines	*Pst*-0 M2, 1-12
Pst-Low	F2	12 lines	*Pst*-I M2, 1-12
Pst-Medium	F2	12 lines	*Pst*-II M2, 1-12
Pst-High	F2	12 lines	*Pst*-III M2, 1-12
F1 *Pc*-Mock	F1	4 lines	*Pc*-0, 1-4
F1 *Pc*-Low	F1	4 lines	*Pc*-I, 1-4
F1 *Pc*-Medium	F1	4 lines	*Pc*-II, 1-4
F1 *Pc*-High	F1	4 lines	*Pc*-III, 1-4
F2 *Pc*-Mock	F2	12 lines	*Pc*-0 M2, 1-12
F2 *Pc*-Low	F2	12 lines	*Pc*-I M2, 1-12
F2 *Pc*-Medium	F2	12 lines	*Pc*-II M2, 1-12
F2 *Pc*-High	F2	12 lines	*Pc*-III M2, 1-12
F1 Salt-Mock	F1	4 lines	S-0, 1-4
F1 Salt-Low	F1	4 lines	S-I, 1-4
F1 Salt-Medium [1]	F1	4 lines	S-II, 1-4
F1 Salt-High [1]	F1	4 lines	S-III, 1-4
F2 Salt-Mock	F2	12 lines	S-0 M2, 1-12
F2 Salt-Low	F2	12 lines	S-I M2, 1-12
F2 Salt-Medium	F2	12 lines	S-II M2, 1-12
F2 S-High	F2	8 lines	S-III M2, 1-12

[1] poor germination and only ~50-100 seeds left.

Table S2. Number of independent t-IR experiments with similar results in matched environments.

Parental Stress	F1 Progeny	F2 Progeny
Pst	3 (1 *Pst*, 2 *Hpa*)	2
Pc	4*	3*
Salt	3	1

*3 and 2 repeats in F1 and F2 progeny, respectively, were only tested in response to the highest stress level.

Table S3. Number of independent t-IR experiments with similar results in mismatched environments.

	Challenge in F1 progeny		
Parental Stress	*Hpa*	*Pc*	Salt
Pst	-	1	1
Pc	3	-	1
Salt	2	2	-
