# Supplementary material for: Costs and Benefits of Transgenerational Induced Resistance in Arabidopsis
Source: Front Plant Sci. 2021 Feb 26;12:644999. doi: 10.3389/fpls.2021.644999 (PMC7952753; doi:10.3389/fpls.2021.644999)
Supplement: Supplementary file 3 [file Data_Sheet_2.pdf]

Supplementary Methods

General growth conditions.

After stratification in water and darkness at 4ºC for 2-3 days, seeds were sown on *Jiffy-7* peat pellets. All plants were initially grown under short-day conditions (8.5 hour -h- light/15.5 h darkness) at 21 °C, 60% relative humidity (RH) and 100-125 µmol s^{-1} m^{-2} light intensity) and watered to saturation by flooding the trays for 0.5 h and removing the excess of water afterwards (3 times/week). After the stress exposure, parental plants (7.5-weeks-old) were moved to long-day conditions (16 h light/8 h darkness) to trigger flowering and set seed. For quantification of disease resistance in F1/F2 populations, plants were grown in individual pots in a randomised block design, and regularly rotated within and between blocks within the climate chamber (3 times/week) to prevent positional effects. For the quantification of NaCl tolerance, agar plates containing multiple F1/F2 populations were rotated with similar frequency.

Microbes and inoculation protocols.

Pseudomonas syringae pv. *tomato* strain DC3000 was cultivated from a frozen glycerol stock for 48 h on King´s B (KB) agar plates, supplemented with 50 µg/ml rifampicin (Sigma-Aldrich, R3501). Cells were collected from agar plates, resuspended and washed in 10mM $MgSO_4$ before adjusting the optical density spectrophotometrically at OD600. For stress induction, cells were adjusted to 5 x 10^7 colony-forming units (CFU)/mL and supplemented with 0.015% Silwet L-77 (Lehle Seeds NC0138454) prior to spraying onto the rosettes of the 4.5-week-old plants until runoff. Mock inoculation was performed by spraying equal amounts of 10mM $MgSO_4$ + 0.015% Silwet L-77). Plants were kept at 100% RH for 1-2 h immediately after inoculation and allowed to recover at 60% RH for 2-3 days before the following inoculation. For quantification of t-IR against *Pst* in F1 progeny, 4.5-week-old plants were challenged by spraying the same bacterial suspension onto the leaves. *Plectosphaerella cucumerina* strain BMM (*Pc*) was cultivated on half-strength Potato Dextrose Agar (BD Difco, BD-213400) for 3.5 weeks in the dark. Spores were resuspended from agar plates in water and filtered through 2 layers of Miracloth (Merck, 475855-1R) to remove mycelium debris. *Pc* inoculum was adjusted to 10^6 spores/ml in water, using a Neubauer haemocytometer. To ensure necrotrophic

infection by the fungus, inoculation was performed by placing 6 µl droplets (10^6 spores/ml) onto fully expanded leaves of approximate similar age; mock inoculum was performed by of applying 6 µl water droplets. For quantification of t-IR against *Pc* in F1 and F2 progeny, 4 leaves/plant of approximate similar age from 4.5-week-old plants were inoculated with *Pc* in a similar manner. The obligate biotrophic Oomycete *Hyaloperonosopora arabidopsidis* strain WACO9 was maintained and bulked on hypersusceptible NahG plants (Ws-0, Syngenta Agribusiness Biotechnology Research, Line 3A). Sporulating plants were collected in 15-mL falcon tubes containing demineralised water and gently shaken to extract conidiospores. The suspension was then filtered through 2 layers of Miracloth and adjusted to 10^5 conidiospores/mL, using a Neubauer haemocytometer. For the quantification of t-IR against *Hpa*, 3-week-old plants were inoculated by spraying the spore suspension onto the shoots, after which plants were maintained at 100% RH.

Analysis of relative growth rate.

Relative growth rate (RGR) analysis was based quantification of green leaf area (GLA) before and after stress treatments. Digital photos (Canon, 500D 15MP) were taken before and after the stress treatment. Digital image analysis of GLA was performed using Adobe Photoshop 6.0. Green pixels corresponding to GLA were selected using a combination of “magic wand” and “lasso” tools and converted into mm^2. For each plant, the following formula was used to calculate RGR, where GLA_2 and GLA_1 are GLA values before and after stress exposure, respectively, and (*t2 – t1*) represents the time-window (d):

$$RGR = \frac{(ln\ ln\ GLA_1)}{(t_2 - t_1)}$$

RGR values were determined for 5- 6 plants per treatment and normalized to the average RGR value of non-stressed plants (mock treatment; 100%). Reproductive fitness was estimated by seed production and seed viability as described in the Supplementary Methods.

Seed production and seed viability assays.

To estimate reproductive fitness, seeds from 5-6 plants per stress treatment were collected in Aracons (Lehle seeds) and weighed. Seed weights for each plant were converted to numbers of seeds, based on the mass of 100 counted seeds (~ 1.2 mg). Seed viability was determined

after sterilisation on agar plates (see for details). Seed viability was quantified on 0.2x Murashige and Skoog (MS) agar (Duchefa, M0221), containing 1% sucrose and 6 g/L agar (pH=5.7, adjusted with KOH). Vapour-phase sterilization was performed by incubating seeds for 4 h in open Eppendorf tubes inside a glass vacuum desiccator (10.5 L), in which 100 ml of bleach (Jantex, R-GG183) and 3 ml of HCl were mixed to produce chlorine gas. Plates were stratified at 4°C in the dark for 3 d and transferred to short-day growth conditions. Seed germination rates were determined in 3-17 replicate plates/population (~25 seeds/plate) at 5 d after stratification. Seeds were considered germinated when green cotyledons were visible.

***Pst* resistance assays.**

After spray-inoculation of 4.5-week-old plants (see above), bacterial growth was quantified at 3 days post inoculation (dpi) by collecting 4 leaf discs/plant in 1.5-mL tubes with 600μL 10 mM $MgSO_4$, using a cork borer (0.75 cm diameter). Leaf discs were homogenised in the tubes using plastic pestles and transferred to 96-wells microtiter plates (Costar®) for serial dilutions in 10mM $MgSO_4$. Twelve samples in each plate were serial-diluted 8 times (5-fold dilutions) and plated onto selective KB agar plates, containing 50mg/mL Rifampicin (Sigma-Aldrich, R3501), using 96-wells Scienceware® replicator (Sigma-Aldrich). For each 96-wells plate, 2 technical replicates were plated onto separate KB agar plates and incubated at 28°C for 2 days before counting CFUs. For each biologically replicated sample (n=10-12), bacterial CFUs were averaged between two technical replicates and 2-3 serial dilutions. For each plant, bacterial CFUs were normalised to its leaf area (mm^2).

***Hpa* resistance assays.**

After spray-inoculation of 3-week-old seedlings (see above), plants were kept at at 100% RH. Shoots were collected at 6 dpi in trypan blue solution (0.067% w/v trypan blue, 33% w/v phenol, 33% v.v glycerol, 33% v.v DL-lactic acid, supplemented with 2 volumes 100% ethanol), boiled for 60 sec, kept at room temperature (RT) for 15 min, boiled again for 30 sec, and incubated at RT for 3 h. Shoots were de-stained in 60% Chloral hydrate (Sigma-Aldrich, 23100) for at least 12 h before scoring. Stained leaves were scored under a stereomicroscope by assigning each leaf to one of the 4 different colonisation classes, which are based on distinct stages of *Hpa* pathogenesis: class I: no hyphal colonisation visible; class II: hyphal colonization without conidiophores; class III: hyphal colonization with conidiophore formation; class IV:

extensive hyphal colonization with conidiophores and sexual oospores. Resistance scoring was based on ~60 seedlings/population (~240 leaves/ population) for F1 plants and ~20 seedlings/population (~80 leaves/population) for F2 plants, representing ~900 leaves/parental stress treatment.

_Pc_ resistance assays.

After droplet-inoculation of 4 leaves/plant (see above), the 4.5-week-old plants were kept at 100% RH and monitored daily for disease progression, which appeared as necrotic lesions surrounded by wider chlorotic halos at the sites of inoculation. Fungal colonisation was quantified by average diameter of the tightly defined necrotic lesion area. The time-point of scoring varied between experiments and was determined when average lesion diameters in a subsample of 5-10 individuals from the control group (progeny of mock-inoculated plants) was >3 mm. Four lesions per plant were averaged and used as unit of statistical replication.

Salt tolerance assays.

Salt tolerance assays were performed as described previously (Verslues *et al.*, 2006; Claeys *et al.*, 2014) with modifications. Seeds were vapour-sterilized, stratified and germinated as described for the seed viability assays. At 5 d after moving the 0.2x MS plates to short-day light conditions, seedlings were transplanted onto new 0.2 x MS plates containing 0 mM, 50 mM or 100 mM NaCl. To avoid contact of leaves with the (NaCl-containing) MS agar, seedlings were positioned along a straight line above which the agar was excised. The root tip was marked on the plate as a reference point to determine root growth. At 5 d after transplantation, the length of the newly formed root from the reference point was determined for each individual plant. Salt stress was quantified as the percentage root growth reduction in each individual plant relative to the average root length on 0 mM NaCl agar of the corresponding line.

Statistical analysis.

Continuous variables were analysed by general linear models. Residuals were first analysed for normal distributions, using Shakiro-Wilk tests and Q-Q plots. If residuals failed to show normal distributions, data were either arcsine-transformed (percentage data), or Box-Cox transformed, using the 'MASS' R package (MASS_7.3-51.5.tar.gz). Models were analysed for

heteroscedasticity, using Levene's tests ('car' R package - car_3.0-0.tar.gz-). When variances were confirmed to be homogeneous, effects of parent treatment were tested by ANOVA models ('nlme' R package - nlme_3.1-137.tar.gz). Statistical significance of parental treatment on F1 resistance against *P. syringae* (colony forming units; CFU) and *P. cucumerina* (lesion diameters; mm) were determined by two separate models. In addition to ANOVA of pooled F1 lines from similarly treated parent plants, using 'aov' function in R base, we performed nested ANOVA with F1 line as random variable, using the 'lme' (method = "REML") and 'anova.lme' function (type = "sequential"; adjustSigma = FALSE) from the 'nlme' R package. Similarly, parental effects on F2 resistance were determined by ANOVA of pooled F2 populations from the same parent plant, as well as nested ANOVA with F2 population as random variable. When both models indicated a statistically significant effect of parent treatment, Tukey HSD post-hoc tests were performed to identify statistically significant differences between pooled F1 populations from similarly treated parent plants or between pooled F2 populations from the same parental ancestor, using the 'TukeyHSD' function in R base. If data continued to show heteroscedasticity after transformation, parental effects were studied by Welch ANOVA, followed by Games-Howell post-hoc tests, using the 'userfriendlyscience' R package (userfriendlyscience_0.7.2.tar.gz). Statistical effects of parental salt treatment on root length (mm) and root length reduction (% relative to 0 mM NaCl treatment) were analysed by two-way ANOVA, in order to separate the effects of parental salt treatment (induction) from progeny salt treatment (challenge). In all cases, type II models were used after having confirmed lack of statistically significant interactions between parent and progeny treatments. The statistical significance of parental effects on root length and root length reduction in F1 plants was determined by 2-way ANOVA of pooled F1 populations from similarly treated parent plants ('aov' function followed by 'Anova' function; type = "II"), as well as 2-way mixed-effect ANOVA with F1 population as random variable ('lmer' function of 'lme4' R package followed by 'Anova' function; type = "II"). Similarly, parental effects on root length and root length reduction in F2 plants were determined by 2-way ANOVA of pooled F2 populations from the same parent plant, as well 2-way mixed-effect ANOVA with F2 population as random variable. When both 2-way ANOVA models indicated a statistically significant effect by parental treatment, pooled F1 populations from similarly treated parent plants were analysed for statistically significant differences at each progeny salt concentration (0 mM, 50 mM and 100 mM), using ANOVA followed by Tukey HSD post-hoc tests. Categorical variables (leaf

frequencies within *Hpa* colonisation classes) were analysed for statistical differences by Fisher's exact tests. Statistically significant effects by parental treatment were assessed by pooling populations derived from similarly treated parents (F1) or a common parental ancestor (F2) in the cross table. Statistical differences between population groups were determined by pairwise Fisher's exact tests after Bonferroni multiple testing correction, using the R package 'fifer' (fifer_1.1.tar.gz).
